# Post-Transcriptional Dynamics is Involved in Rapid Adaptation to Hypergravity in Jurkat T Cells

**DOI:** 10.3389/fcell.2022.933984

**Published:** 2022-07-04

**Authors:** Christian Vahlensieck, Cora S. Thiel, Daniel Pöschl, Timothy Bradley, Sonja Krammer, Beatrice Lauber, Jennifer Polzer, Oliver Ullrich

**Affiliations:** ^1^ Institute of Anatomy, Faculty of Medicine, University of Zurich, Zurich, Switzerland; ^2^ Innovation Cluster Space and Aviation (UZH Space Hub), Air Force Center, University of Zurich, Dübendorf, Switzerland; ^3^ Space Life Sciences Laboratory (SLSL), Kennedy Space Center (KSC), Merritt Island, FL, United States; ^4^ Space Biotechnology, Department of Machine Design, Engineering Design and Product Development, Institute of Mechanical Engineering, Otto-von-Guericke-University Magdeburg, Magdeburg, Germany; ^5^ Space Medicine, Ernst-Abbe-Hochschule (EAH) Jena, Department of Industrial Engineering, Jena, Germany; ^6^ Zurich Center for Integrative Human Physiology (ZIHP), University of Zurich, Zurich, Switzerland

**Keywords:** immune cells, gravity-sensing, hypergravity, space flight, altered gravity, gene expression

## Abstract

The transcriptome of human immune cells rapidly reacts to altered gravity in a highly dynamic way. We could show in previous experiments that transcriptional patterns show profound adaption after seconds to minutes of altered gravity. To gain further insight into these transcriptional alteration and adaption dynamics, we conducted a highly standardized RNA-Seq experiment with human Jurkat T cells exposed to 9xg hypergravity for 3 and 15 min, respectively. We investigated the frequency with which individual exons were used during transcription and discovered that differential exon usage broadly appeared after 3 min and became less pronounced after 15 min. Additionally, we observed a shift in the transcript pool from coding towards non-coding transcripts. Thus, adaption of gravity-sensitive differentially expressed genes followed a dynamic transcriptional rebound effect. The general dynamics were compatible with previous studies on the transcriptional effects of short hypergravity on human immune cells and suggest that initial up-regulatory changes mostly result from increased elongation rates. The shift correlated with a general downregulation of the affected genes. All chromosome bands carried homogenous numbers of gravity-sensitive genes but showed a specific tendency towards up- or downregulation. Altered gravity affected transcriptional regulation throughout the entire genome, whereby the direction of differential expression was strongly dependent on the structural location in the genome. A correlation analysis with potential mediators of the early transcriptional response identified a link between initially upregulated genes with certain transcription factors. Based on these findings, we have been able to further develop our model of the transcriptional response to altered gravity.

## Introduction

The immune system is among the biological systems that react the most to disturbances during spaceflight (Choukèr and Ullrich, 2016; Frippiat et al., 2016). There are several known cases of immune system-associated health problems during ISS missions (Mehta et al., 2013; Crucian et al., 2016a; Crucian et al., 2016b; Kunz et al., 2017), an effect that could potentially limit humankind’s ability for long-term exploratory-class missions (NASA Office of Inspector General, 2018). Aside from classical factors resulting in a reduced immune defense like stress and increased radiation, microgravity has a direct influence on various defense abilities of immune cells (Sonnenfeld, 2002; Crucian et al., 2018). The negative effects on adaptive immunity persist during long-term missions (Crucian et al., 2015) and affect several cell types, which has been demonstrated in independent studies (Kaur et al., 2005; Kaur et al., 2008; Crucian et al., 2014). Among other affected cell types, human T cells display several changes on multiple levels including gene expression (Chang et al., 2012), chromatin regulation (Paulsen et al., 2010), epigenetics (Singh et al., 2010), cell cycle regulation (Thiel et al., 2012), micro-RNA expression (Mangala et al., 2011), apoptosis regulation (Lewis et al., 1998; Battista et al., 2012) and cytokine IL-2/IFN*g* expression (Cogoli and Cogoli-Greuter, 1997).

More recently, it was discovered that initial effects of altered gravity already appear after seconds of exposure. The production of reactive oxygen species from macrophages stops almost immediately after onset of microgravity and recovers after ∼40 s (Adrian et al., 2013; Thiel et al., 2017a). Another early effect acts on the transcriptome, which has been described to be affected after only 20 s during parabolic flights. A seemingly random distribution of genes showed significantly different expression levels after this short period of time. In parallel, a comparable effect has been described for exposures to 75 s of hypergravity and 5 min of microgravity in transcriptomics experiments during sounding rocket campaigns (Thiel et al., 2017b; Thiel et al., 2018). After 75 s of hypergravity, 84.4% (Jurkat T cells) of transcript levels with initially altered expression returned to standard gravity levels. Furthermore, almost 100% (Jurkat T cells) of initially altered gene expression levels adapted to the new gravitational environment after 5 min of microgravity. Concomitantly, a second “transcript pool” appeared, constituted of genes that were initially unaltered (20 s) but displayed changed expression levels after minutes of altered gravity (Thiel et al., 2017c; Thiel et al., 2017d). Some genes were described as stably expressed in all gravitational environments analyzed, including the well-known reference genes ABCA5, GAPDH, HPRT1, PLA2G4A, and RPL13A, but also olfactory genes located in the chromosomal region 11p15.4, as demonstrated in U937 cells. Nevertheless, this only affects a small minority of genes, no systematic commonalities are known on the level of single genes.

Gene expression studies are a frequently used tool to perform whole genome analyses in altered gravity. A wide variety of tissues and cell types are used often originating from rodents and humans, but also from *Xenopus*, *Drosophila*, yeast, bacteria, plants, and other species (Clement, 2012:; Najrana and Sanchez-Esteban, 2016). These studies showed that a plethora of different genes are up-regulated and down-regulated. However, variable and contradictory results are common, but an overall evidence indicates that many functions such as signal transduction, cell-cell contact, cytoskeleton, and cell migration tend to be altered in microgravity (Clement, 2012). A general mechanism of how the altered gravity is transduced into the cell and causes altered gene expression has not yet been finally proven. Because changes in mechanical forces (to which gravity forces also belong to) can be sensed throughout the entire cell and transported by mechanotransduction across the cytoskeleton into the cell and into the nucleus, the gravitational signal could be propagated to the chromatin and cause changes in gene expression through changes in the spatial position of the chromatin (Najrana and Sanchez-Esteban, 2016; Uhler and Shivashankar, 2017). Indeed, in a recent study, we were able to show that genes were up- or down-regulated in altered gravity in structural genome clusters in human Jurkat T cells that we defined as gravity-responsive chromosomal regions (GRCRs). Based on the combination with an additional high-throughput chromatin conformation capture (Hi-C) analysis we could show that these one-dimensional chromosomal regions overlap with and correspond to the Hi-C identified 3D chromatin structural changes. These regions colocalize with differential chromatin conformation interactions on the small chromosomes (chr16-chr22). With this study we found first evidence that changes in the gravitational force are transmitted into the nucleus where they induce 3D chromosomal conformational changes which are associated with a rapid transcriptional response in immune cells (Vahlensieck et al., 2021b).

One main obstacle in model refinement is the complex response of the cell to altered gravity, happening on various layers (Thiel et al., 2017d). The highly sensitive differential transcriptomic response in particular is composed of 1000s of differentially expressed genes (Thiel et al., 2017b), which renders it difficult to pinpoint mechanistical actors. We could recently demonstrate the power of cross-campaign correlation studies in tracking the time course of gene expression (Vahlensieck et al., 2021a). However, the comparability between most altered gravity studies is limited by the fact that different experimental designs, platforms, and experimental procedures have been used. Campaigns and platforms are limited in terms of maximum time windows, sample capacity and statistical reproducibility by biological replicates versus covering multiple conditions. For comparative studies, ground-based facilities (GBFs) offer highly comparable conditions and can cover large time ranges. GBFs are technical instruments that try to emulate the altered gravity environment in a ground environment and are mostly easily available. For microgravity, several approaches are known including clinostats, random positioning machines (RPMs), rotating wall vessels and magnetic levitation. Except for the latter, they are all based on averaging out the gravity vector over time. Based on the theory of the gravity continuum, a systematic investigation of the range <1xg to approximately 0xg would be obvious. While operational constraints in real microgravity flight samples do not allow comparability at the level of maximum standardization, significant methodological limitations exist for ground-based simulations, too (Bevelacqua et al., 2020). Previous studies of ground-based 0xg simulations using the RPM showed significant shear forces (Wuest et al., 2015; Hauslage et al., 2017), which can reach up to 100 mPa (Wuest et al., 2017). Also, the central basis of the clinostat principle, has been questioned: That zero gravity corresponds a vector sum zero of acceleration force, may be true for a point particle, but not for an extended system (Bevelacqua et al., 2020). Indeed, in our own previous experiments with human Jurkat T cells, less than 1% of the transcriptome changes in flight-induced microgravity could be reproduced by clinorotation (Thiel et al., 2020). For this reason, the existing ground-based simulations did not appear to us to constitute a methodologically acceptable experimental basis for the systematic study of Jurkat T cells in altered gravity.

For hypergravity however, centrifuges are a reliable alternative that only have small deviations from linearly applied hypergravity. In terms of transcriptomics technology, RNA-Seq supports further analyses than quantitative gene expression: The sequencing of reads from poly-A RNA-Seq libraries regularly and robustly covers intronic, unspliced regions (Kapranov et al., 2010). This allows for separate modelling and quantification of the unspliced and the spliced counts of the transcript pool of a gene. Since unspliced transcripts will be consecutively spliced, the fraction of unspliced counts is predictive of the spliced counts, which is exploited for RNA velocity analysis (La Manno et al., 2018). This prevalent information in RNA-Seq datasets can be used to separate differentially expressing genes into those that freshly expressed and those where the transcripts are older (Zeisel et al., 2011). Further, it can be used to differentiate between pre- and post-transcriptional regulation, i.e. effects that only affect the spliced pool must appear post-transcriptionally (Gaidatzis et al., 2015).

The underlying mechanism of cellular graviperception is not known to date. The cell itself is potentially not heavy enough to sense a direct effect of gravity, yet the membrane including surrounding medium could be (Albrecht-Buehler, 1991). There are considerations about a direct mechanical effect on cell shape and geometry (Häder et al., 2017) that is propagated from the cellular membrane *via* the cytoskeleton into the entire cell, including the nucleus (Vorselen et al., 2014). Recently, we discovered that short-term gravitational effects on the transcriptome are conserved on the level of genomic regions instead of single genes, which colocalized with differential chromatin conformation on small chromosomes chr16-chr22, except for chr18 (Vahlensieck et al., 2021b). Based on these structural insights, we were able to formulate an initial model where altered gravity acts on the cell membrane, propagates *via* the cytoskeleton into the nucleus, altering its shape and thereby rearranging the chromatin. This leads to transcriptional activation and transcriptional repression of certain areas of the chromatin, which results in differentially expressed areas. Yet, the model remains highly preliminary. The more knowledge about gravity-induced effects is available, the more concrete the hypothesis can be refined, increasing our understanding of potential mechanisms of gravisensing. In this regard, transcriptomics is an ideal technique due to its easy experimental accessibility paired with a high density of information.

Concludingly, to support detailed model building, we realized the necessity of a highly standardized, multiple-timepoint transcriptomics study from a single platform including spliced and unspliced read discrimination. This would allow for temporal comparisons fully mitigating potential cross-platform effects and would be highly beneficial to enhance our understanding of transcriptional effects over time.

## Materials and Methods

### Preparation of Biological Samples

Samples were generated, processed and sequenced as described previously (Vahlensieck et al., 2021a). Briefly, Jurkat T cells (ATCC Manassas, United States, Clone E6-1, TIB152™) were cultured in RPMI 1640 (Biochrom, Berlin, Germany Cat. Nr FG1215) medium, supplemented with 10% FCS and 1% Pen/Strep, without centrifugation steps. The concentration was adjusted to 5 × 10^6^ cells/mL, samples were transferred into sterile, prewarmed (36.5°C) 2 ml pipettes and centrifuged using a custom-built 9xg pipette centrifuge provided by KEK (Bad Schmiedeberg, Germany). 10 samples per condition were generated. Pipettes were drained into 5 ml sterile, RNAse/DNase-free plastic tubes. RNA was extracted, poly(A) enriched with the help of the NEBNext Poly(A) mRNA Magnetic Isolation Module, library prepped with the NEBNext Ultra II RNA Library Prep Kit, both from New England Biology (Ipswich, United States), and sequenced (RNA-Seq) with appropriate quality control and standardization. There were three conditions, one control condition with 15 min at 1xg, one with 15 min at 9xg and one with 12 min at 1xg plus 3 min at 9xg (see results part for the underlying rationale).

### Canonical RNA-Seq Pipeline

Raw reads were processed as described previously (Vahlensieck et al., 2021a). For the pipeline workflow, compare Supplementary Figure S1.

### Transcriptional Dynamics Analysis

Observable (adj. *p*-value not NA) genes were characterized as upregulated (adj. *p*-value < 0.05, LFC >0), downregulated (adj. *p*-value < 0.05, LFC <0) or not significantly changed (adj. *p*-value > 0.05), for each contrast of adjacent time points (hypg3-Ctrl, hypg15-hypg3). Genes in the grey balls at 15 min are initially downregulated/upregulated and later either constant (inner grey balls) or upregulated/downregulated (outer grey balls) but still lower/higher than the control.

### Spliced and Unspliced Differentially Expressed Genes

For Velocyto (La Manno et al., 2018), “LOOM_NUMERIC_DTYPE” in constants.py was changed from “UInt16” to “UInt32”. Generic “CB”/“UB” tags were added for each read *via* Simplesam. The Velocyto command line interface (CLI) version 0.17.17 was used (-U: without umi, -c: one file per cell). Single loom output files were obtained per input bam (one bam file per sample), from which respective spliced/unspliced count levels (layers) were extracted using Loompy. DESeq2-mediated Differential Expression (DE)-calling was performed per level using identical count normalization factors, calculated on the sum of counts from spliced, unspliced and ambiguous levels. For the pipeline workflow, compare Supplementary Figure S1.

### Chromosomal Differentially Expressed Gene Distribution

Chromosomal DEG distribution analysis was performed as described previously (Vahlensieck et al., 2021a). The analysis was repeated on the level of chromosome cytobands (first level, 299 bands). Spearman correlation coefficients were calculated between the vector of expected numbers and the vector of actual numbers, for absolute numbers of DEGs and up/down distribution.

### Differential Exon Usage Analysis

DEU analysis was performed as described previously (Vahlensieck et al., 2021a). For the pipeline workflow, compare Supplementary Figure S1. Intron retention analysis was performed with IRFinder with standard settings.

### Exon Usage Dynamics–Chromosomal Distribution

Significantly differentially used exons and corresponding genes were obtained from DEXSeq and the exon log fold change between conditions was used to identify exons with in-/decreased usage. Exons with in-/decreased usage and genes with significant DEU were mapped to chromosomes *via* BiomaRt using associated genes. Fisher’s exact test was used as described for chromosomal DEG distribution. Expected counts of exons with higher usage were calculated by multiplying the observed number of differentially used exons per chromosome with the global ratio of higher to lower exon usage.

### Exon Usage Dynamics–Transcript Biotypes of DU-Exons

DEXSeq result data was filtered for exon-level, FDR-adjusted *p*-values below 0.05. In case a DU-exon mapped to multiple transcripts, all of these were included in the subsequent analysis. Duplicated transcripts were removed, while a single instance of each was retained, yielding a qualitative representation of transcripts affected by DEU. Finally, remaining transcripts were mapped to Ensembl transcript biotypes using biomaRt. These included protein-coding, transcripts that are usually translated; retained introns, transcripts that could still be translated but could be affected by complex regulation patterns; ORF-lacking (Ensembl: “processed transcripts”), that do not contain an open reading frame (ORF) and are not translated; nonsense-mediated decay, carrying an early stop codon that marks them for degradation; and lncRNA.

### Exon Usage Dynamics–Patterns in Differential Exon Usage-Transcript Biotypes and Differentially Expressed Gene-regulation

To investigate potential associations between altered exon usage- and differential gene expression, DEU-transcripts were processed as described in “Transcript biotypes of DU-exons” and additionally mapped to corresponding genes. Up-/downregulated genes were selected by DESeq2 LFC above/below 0 and FDR-adjusted DESeq2 *p*-value below 0.05. For each unique DEU-transcript biotype, DEGs without corresponding DEU-transcript were removed, respectively. DEU-transcript biotype matching DEG groups containing less than 20 DEGs were excluded from further analysis for clarity and minimization of multiple testing correction. Next, Fisher’s exact test was applied on DEU-transcript-filtered DEG groups stratified by unique DEU-transcript biotype, to reveal whether DEGs associated with a specific DEU-transcript biotype show different regulation to the background (Fisher test: DEGs up/down, this/other biotypes). The above was carried out within each contrast and splicing subset separately. Obtained *p*-values were FDR-adjusted and results below 0.05 considered significant. Expected counts of downregulated DEGs within each DEU-transcript-biotype DEG group were calculated by multiplying the total number of DEGs of the group with the downregulated fraction among all DEGs within the respective contrast and splicing subset.

### Protein Coding Counts Ratio

Fastq files were pseudoaligned against hg38 with Salmon in subsampling mode. Normalized counts were generated with Sleuth. Transcripts were mapped to Ensembl transcript biotypes using biomaRt. Counts were summed per gene per condition and split into counts for noncoding transcripts (intron retention + ORF-lacking + nonsense-mediated decay) and counts for coding transcripts. For each condition, the transcript biotype ratio was calculated by dividing the counts of coding transcripts by the sum of counts of coding and noncoding transcripts. If the ratio changed between Ctrl and hypg3 by more than one percent point, a gene was called increased PCCR if positive and decreased PCCR if negative, otherwise constant PCCR. For these subsets of genes, the number of differentially expressed genes (standard/spliced/unspliced) was then calculated by counting the number of genes that had an FDR-corrected *p* value <0.05.

### Gene Set Enrichment Analysis

Fast preranked gene set enrichment analysis [FGSEA (Sergushichev, 2016)] was used against hallmark, gene transcription regulation database (GTRD) target and gene ontology gene sets. Genes were preranked using the DESeq2 stat parameter, and FGSEA’s eps variable was set to zero. Gene sets with more than 400 annotations or *p*-values above 10^−5^ were excluded. Pathways were collapsed. Obtained matrices were merged and gene sets with highest absolute, normalized enrichment score were used for heatmap visualization.

### Compartmentalization Analysis

Gene lists for which transcripts were enriched in stress granules and P bodies were used, where stress granules/P bodies had been purified and consequently sequenced by RNA-Seq (Matheny et al., 2019).

Lists were filtered for genes detected in this study. For each temporal comparison, the number of significantly up- and downregulated genes with compartment enrichment was calculated. Fisher’s exact test was used for overrepresentation analysis, FDR cutoff of 0.05.

## Results

**FIGURE 1:**
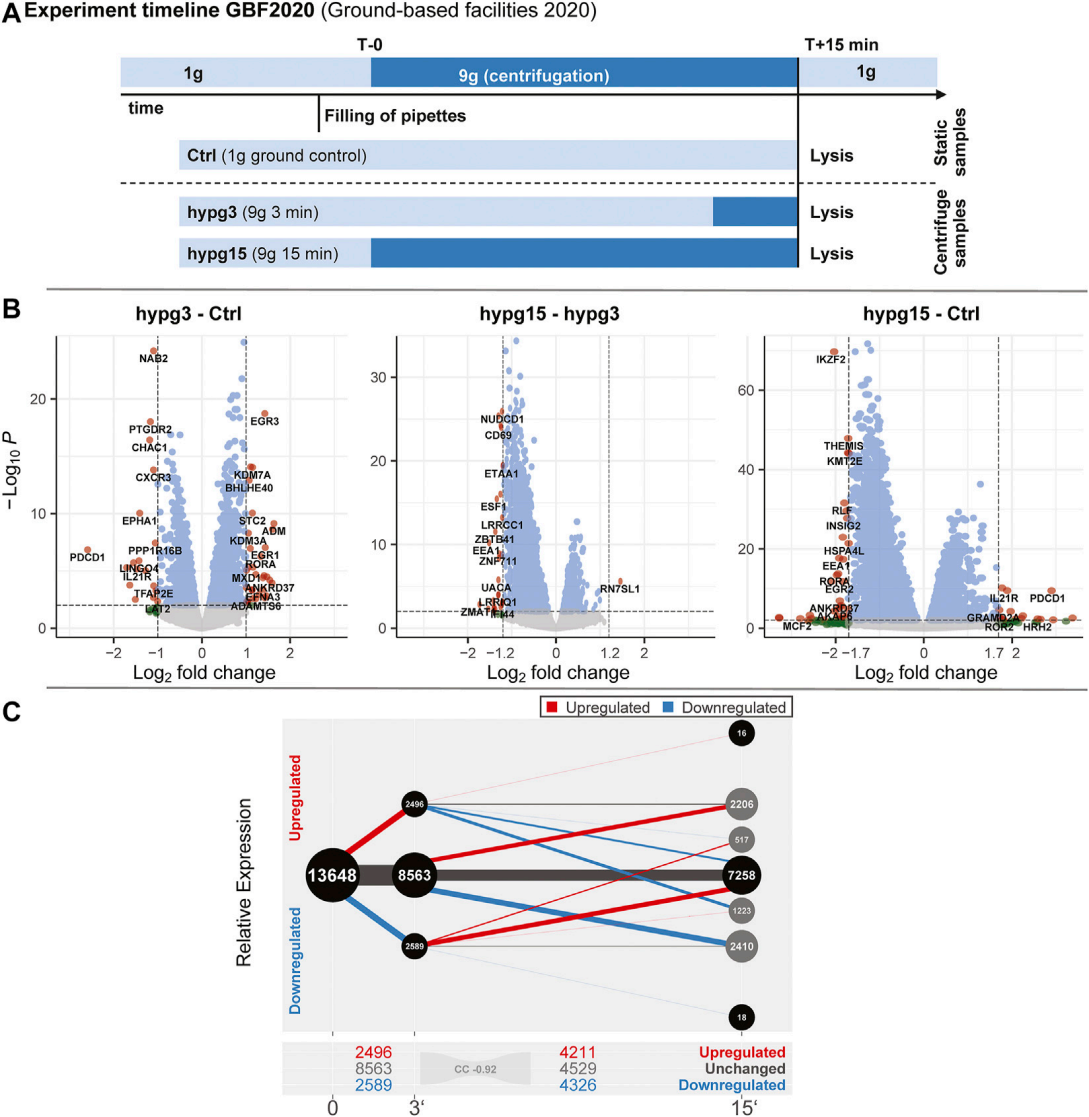
Global dynamics of the effects of hypergravity on the transcript pool. **(A)** Experiment fixation scheme. Overview of the experimental conditions for which samples were acquired. During the ground-based facilities 2020 campaign, Jurkat T cells were filled into 1 ml pipettes, incubated for 15 min and then rapidly emptied into RLT lysis buffer. During these 15 min, four samples remained at 1xg gravity (Ctrl), and four samples stayed at 1xg for 12 min and where consequently exposed to 9xg on a pipette centrifuge for 3 min before lysis (hypg3). A third group was exposed to 9xg for the entire 15 min before lysis (hypg15). **(B)** Volcano plots of all three temporal comparisons show the overall distribution. Genes with highest fold change that exceed the *p* value thresholds of ±1.0/±1.2/±1.7 are annotated. Hypg15-hypg3 and hypg15-Ctrl show a pronounced skew towards downregulation, for hypg3 a slight tendency towards upregulation is apparent. An MA plot analysis can be found in Supplementary Figure S3. **(C)** Temporal coherence of gene expression. The number of genes that were upregulated/downregulated after 3 min is shown (three balls at 3′). Starting from these pools, genes that were consistently or even exceedingly up-/downregulated (lines to upper two balls, lower two balls), those that were no longer differentially regulated (lines to center ball) or only slightly elevated/lower (lines to inner two grey balls), or even regulated in the opposite direction (lines crossing the middle) are displayed. The line width represents the number of genes in each category. Most genes that were differentially expressed after 3 min were either no longer differentially expressed (dominantly for downregulated) or even regulated in the opposite direction (dominantly for downregulated) after 15 min. Continuous regulation in a single direction was very rare. The Spearman correlation coefficient between fold changes of the hypg3-Ctrl and the hypg15-hypg3 comparison is displayed at the bottom.

The aim of this study was to compare the effects of hypergravity on human Jurkat T cells to improve our understanding of cellular transcriptional dynamics caused by altered gravity. Importantly, such a study should ensure maximum comparability, allow for high number of replicates, and yield maximum information for the system under study. This was achieved by conducting a ground-based facility (GBF) campaign on a 9xg pipette centrifuge [compare (Thiel et al., 2017c)]. We chose two time points of hypergravity, one at 3 min, which is the shortest global transcriptional stress response described for heat shock experiments in mammalian cells (Mahat et al., 2016). Furthermore, this time point represents early effects covered by the short-term altered gravity platforms such as parabolic flight (20 s hypergravity and microgravity) and suborbital ballistic rocket (75 s hypergravity, 5 min microgravity) (Thiel et al., 2017b) experiments. The other time point was chosen at 15 min, where adaptive processes already occur compared to early-stage effects [as described in (Vahlensieck et al., 2021a)]. The experiment consisted of three sample groups (Figure 1A): 1) 1xg control (Ctrl) 2) 9xg for 3 min (hypg3), 3) 9xg for 15 min (hypg15). For each sample group, four samples were processed and analyzed by RNA-Seq (polyA-enriched library, 25M reads, 75 bp, paired ends, details in methods section). From these three sample groups, each containing four RNA-Seq samples, three temporal comparisons could be generated: One for assessing initial effects (hypg3-Ctrl), one for later stage effects (hypg15-hypg3) and one for evaluating residual effects over the entire timespan (hypg15-Ctrl).

### Global Transcriptional Dynamics Reveal a Temporal Rebound Effect

At first, we assessed the general differential gene expression behavior of the different sample groups. The RNA-Seq transcriptomics data were analyzed with a standard featureCounts- and DESeq2-based pipeline (Supplementary Figure S1, red boxes). The different gravity conditions allow sharp clustering of the transcriptomics samples within their condition groups (Supplementary Figure S2), as visualized by the top 30 genes with highest mean variance (Supplementary Figure S3A). This clear separation highlights the impact of altered gravity on cellular transcriptomics.

First, we investigated the overall regulation patterns which included a plethora of genes (volcano plot in Figure 1B, MA plot in Supplementary Figure S3B). The fold change distribution showed a slight shift towards upregulation for the genes with high absolute fold changes in the hypg3-Ctrl comparison. Most fold changes are below ×2, which is not unexpected considering the short exposure time frame (compare discussion). Between 3 and 15 min, a strong skew towards downregulation could be observed. Similarly, comparing 15 min versus control, downregulation is dominant for differentially expressed genes (Figure 1B).

To better assess these effects and to understand the behavior of differentially expressed genes in different comparisons, a temporal coherence analysis was conducted. For each gene, the qualitative expression change after 3 min (i.e., significantly downregulated, upregulated, not differentially expressed) was matched with the change after 15 min (Figure 1C). The majority of differentially expressed genes (DEGs) showed not only adaptation as previously described for Jurkat T cells (Thiel et al., 2017b) but an inversion, so far only postulated for U937 macrophage cells between 20 and 75 s hypergravity resp. 5 min microgravity (Thiel et al., 2018): Genes that were initially upregulated after 3 min were mostly downregulated after 15 min or not differentially expressed anymore. Genes that were initially downregulated were subsequently either not differentially expressed anymore or upregulated. Remarkably, a continuous regulation with same direction after 15 min appeared in less than 10% of genes. Additionally increased expression appeared only for 34 genes. Additionally, in a second-phase response, many genes that were not differentially expressed after 3 min were up- or downregulated after 15 min, as expected from prior studies (Thiel et al., 2017b). This temporal inversion was termed transcriptional rebound effect since differential expression shifted to the opposite direction for many genes at the second point in time. It was further quantified by computing a Spearman correlation coefficient of -0.92 between fold changes after 3 min versus fold changes between 15 and 3 min, indicating strong anticorrelation. We further analyzed if the small fraction of continuously regulated genes might correspond to a functional involvement (Supplementary Figure S4). Only an enrichment of 22 upregulated genes in chromatin organization and downregulation of around 30 genes in mitochondrial ATP synthesis emerged as potentially functional relevant.

### Differential Expression is Highly Asymmetric Between Spliced and Unspliced Transcripts

The short periods between the sample timepoints do not allow much time for transcription, splicing and degradation of mRNA, especially for the comparison hypg3-Ctrl. Considering the strong differences between the timepoints and the skewed volcano plots, the cells appeared to be in a highly dynamic transcriptional state having not yet reached a new steady state. Transcription and consecutive processing of novel transcripts requires up to 10 min for the average gene (Phillips and Milo, 2016). This exceeds the hypergravity incubation time of 3 min by far, therefore genes that started transcription at the onset of hypergravity likely are not spliced when the samples are fixed. We tried to separate the datasets between potentially older and potentially freshly transcribed RNA to gain better insights into these early dynamics. From RNA velocity analysis, it is known that the concentration of unspliced transcripts approximates the future concentration of spliced transcripts since freshly transcribed RNA appears first in the unspliced pool before appearing in the spliced pool (La Manno et al., 2018). Therefore, we separated potentially newer unspliced from potentially older spliced transcripts. For this purpose, the single cell RNA-Seq pipelines Velocyto and Loompy were adapted for bulk RNA-Seq data to quantify spliced and unspliced fractions. After separation, the fractions were analyzed separately in DESeq2, like in the standard featureCounts pipeline (Supplementary Figure S1 violet boxes). This novel approach required thorough validation against the standard RNA-Seq pipeline which demonstrated the validity of the results (Supplementary Figures S5, S6).

**FIGURE 2:**
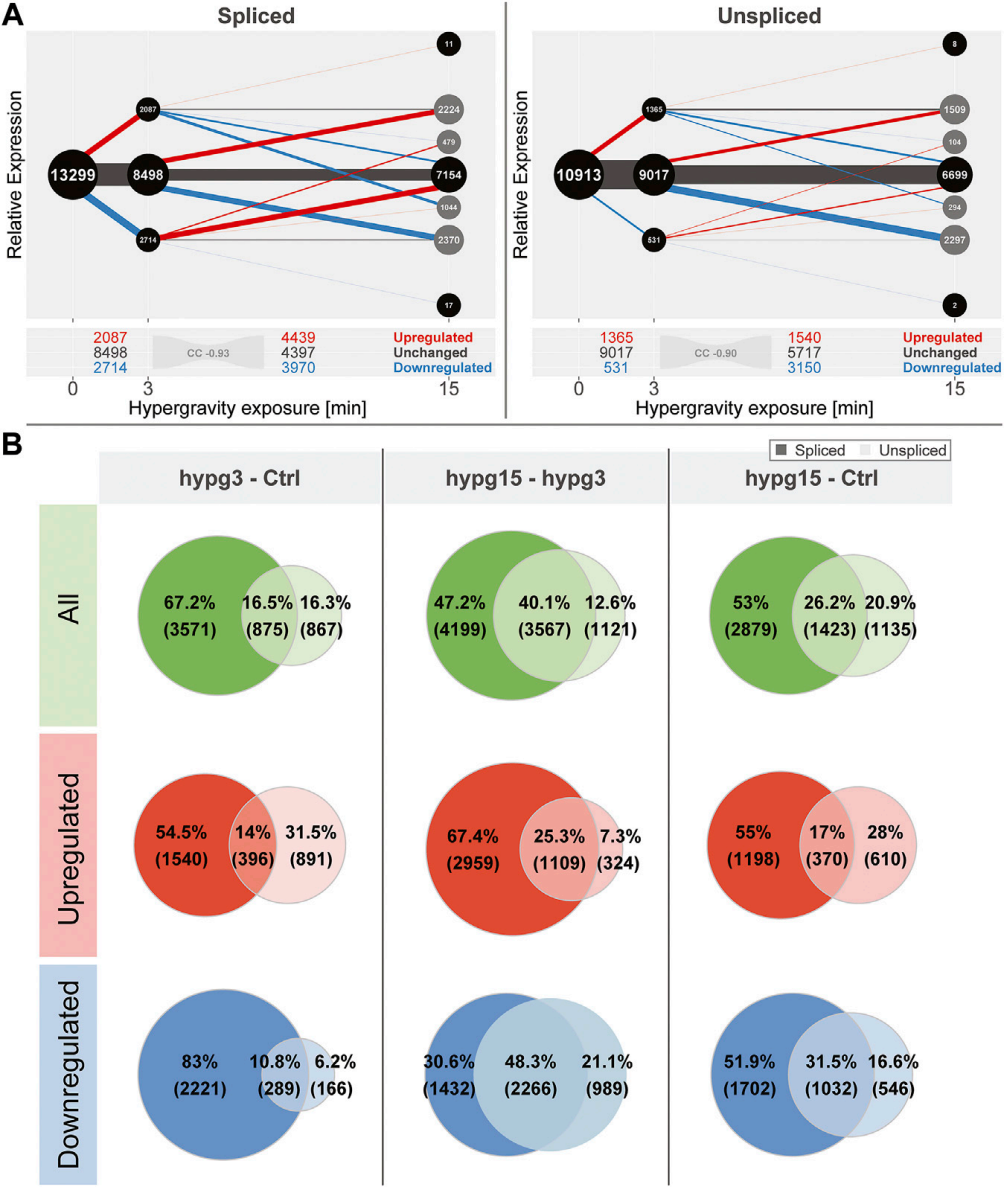
Global dynamics of the effects of hypergravity on the spliced and unspliced transcript pool. **(A)** Temporal coherence of gene expression in the spliced and unspliced pool, analogously to Figure 1C. Again, the fraction of genes that were consistently regulated in one direction was very small. The initial regulation pattern after 3 min was not symmetric anymore but showed a preference for downregulation for the spliced fraction and a strong preference for upregulation for the unspliced fraction. **(B)** Overlap between spliced (strong colors) and unspliced (light colors) fractions for all genes, only upregulated, and only downregulated genes for all three temporal comparisons. The fraction of unspliced differentially expressed genes (DEGs) was small compared to spliced DEGs after 3 min and became larger in the following comparisons. This was mostly driven by the low number of downregulated unspliced genes after 3 min but not by upregulated genes after 3 min.

When conducting the temporal coherence analysis from Figure 1C, strikingly different results emerged for the spliced and the unspliced fraction (Figure 2A). Consistently, both again showed a transcriptional rebound effect with a correlation coefficient of fold changes of −0.93 for spliced and −0.90 for unspliced. However, spliced transcripts showed a stronger tendency towards downregulation after 3 min, whereas unspliced transcripts showed much more dominant upregulation after 3 min and a strong tendency towards downregulation between 3 and 15 min. No tendency towards a specific transcript biotype could be detected, neither for the standard analysis, nor for the split between spliced and unspliced genes (Supplementary Figure S7).

Not all genes that were detected in the unspliced fraction could be detected in the spliced fraction and vice versa. This is most likely due to several effects: the split into spliced and unspliced transcripts corresponds to the ratio of intron-bearing reads to those where two exons are directly adjacent to each other and therefore intron-less. If a gene did not carry any introns in the genome, it appeared per definition in the spliced pool immediately after transcription. Additionally, since the experiment was based on polyA-enrichment of RNA, the fraction of unspliced transcripts was smaller. Consequently, some genes would be detected in the spliced fraction but not in the unspliced if they fell below the count filtering threshold of DESeq2. Because of different transcription, splicing and degradation kinetics, some genes might also only accumulate spliced or unspliced transcripts since the other species is quickly processed or degraded. To focus on relations between the pool of unspliced and spliced genes, an analysis of the subset of genes that can be quantified in both fractions was performed (Figure 2B). The overlap of differentially expressed genes in the spliced and the unspliced fraction was quantified for all genes, only for upregulated genes, and only for downregulated genes for all three temporal comparisons, respectively. The fraction of genes that were called differentially expressed on the unspliced level increased over time. This was almost exclusively driven by downregulated genes: they accounted for only 17% of differentially expressed genes (DEGs) after 3 min, including overlaps with spliced, but rose to 69.4% of DEGs between 3 and 15 min. The ratios of upregulated genes in the unspliced fraction were more stable over time.

Given these numbers, hypergravity up- and downregulated unspliced and spliced transcripts reacted differently: In the first 3 min, upregulation affected both unspliced and spliced transcripts, in line with active novel transcription. On the other hand, downregulation did initially not affect unspliced transcripts, suggesting active degradation rather than halted transcription. These differences seemed to equalize between 3 and 15 min of hypergravity.

### Transcriptional Changes in Hypergravity Affect the Entire Genome With Non-random up- and Downregulation

**FIGURE 3:**
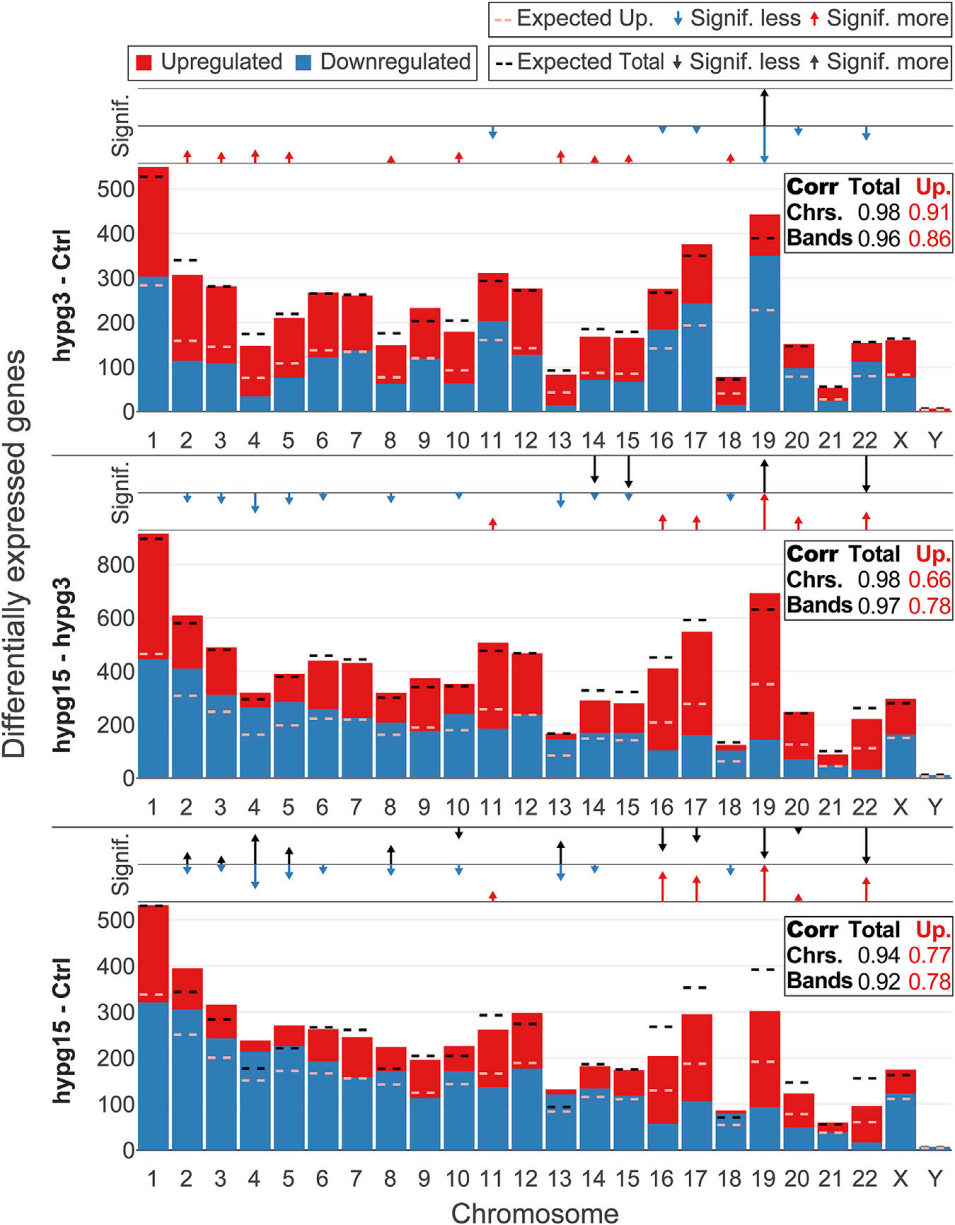
Chromosomal distribution of differential gene expression. Number of differentially upregulated (red) and downregulated (blue) genes per chromosome (horizontal axis) for all three comparisons. The expected number of total DEGs per chromosome (based on the fraction of differentially expressed genes for all genes and the number of detected genes per chromosome and assuming a uniform probability of differential expression) is shown as a black dashed line, the expected number of upregulated genes out of up- and downregulated genes is shown as dashed light red line. Above each diagram, arrows show if the actual number of all DEGs (upper row)/upregulated genes (lower row) lies significantly above (arrow pointing upwards, arrow length represents magnitude of deviation) or below (arrow pointing downwards, arrow length represents magnitude of deviation) the expectation. In the box on the right, the correlation coefficient between the expected and the actual number of total DEGs/upregulated genes is shown, both on the chromosome level (as displayed in the diagram) and on the chromosomal cytoband level. The closer the correlation coefficient comes to 1, the more the actual number corresponds to the expected number. Initially, DEGs were evenly distributed over all chromosomes, tightly following the expectation, but upregulation versus downregulation was non-evenly distributed between chromosomes. Later in time, also absolute numbers no longer corresponded to the expectations. The same figure split by chromosome cytobands can be found in Supplementary Figure S8.

As a next step it was investigated whether differential expression preferably affected genes in a certain genomic region, which could point towards a mechanism or pathway. Therefore, differentially expressed genes were mapped to their harboring chromosomes (Figure 3) and their corresponding chromosomal cytobands (Supplementary Figure S8), split by up- and downregulated genes for all three temporal contrasts. For the absolute number of DEGs per chromosome and for the ratio between up- and downregulated genes per chromosome, the value expected from random drawing from a uniform distribution was compared to the actual numbers. Additionally, a correlation coefficient was calculated between expected and actual numbers for the absolute number of DEGs and for the ratio between up- and downregulation. These analyses were also conducted on the chromosome cytoband level (Figures 3 tables labeled “corr”).

After 3 min, the overall number of DEGs per chromosome was near the expectation, leading to a correlation coefficient (CC) of 0.98. Only chromosome 19 carried significantly more DEGs than expected, at an FDR-corrected *p* value of 0.018. For the cytobands, this was also the case (CC of 0.96). For the relative number of up-versus downregulated genes, however, the situation was very different: 10/24 chromosomes carried more upregulated DEGs than expected and 6/24 more downregulated genes, with chromosome 19 displaying the highest skew towards downregulation. Between 3 and 15 min, this trend was inverted, in line with the previously described rebound effect: Chromosomes that previously tended towards more upregulation now tended towards more downregulation, and *vice versa*. Further, the number of chromosomes that significantly differed in terms of the total number of DEGs was elevated at 4 instead of 1. Again, chromosome 19 was the most affected and harbored many more upregulated relative to downregulated genes than expected. For the comparison hypg15-Ctrl, there were almost as many chromosomes with absolute numbers significantly different from the expectation as there were chromosomes with significantly different up/down ratios.

In summary, differential gene expression was initially distributed over the entire genome with no local aggregation but showed strong skews in terms of up-versus downregulation. Over time, the absolute numbers progressively deviated from the expectation assuming a uniform distribution, but the skew in up-versus downregulation remained. Altered gravity therefore affected transcription throughout the entire genome, but the direction of differential expression was heavily dependent on the genomic location.

**FIGURE 4:**
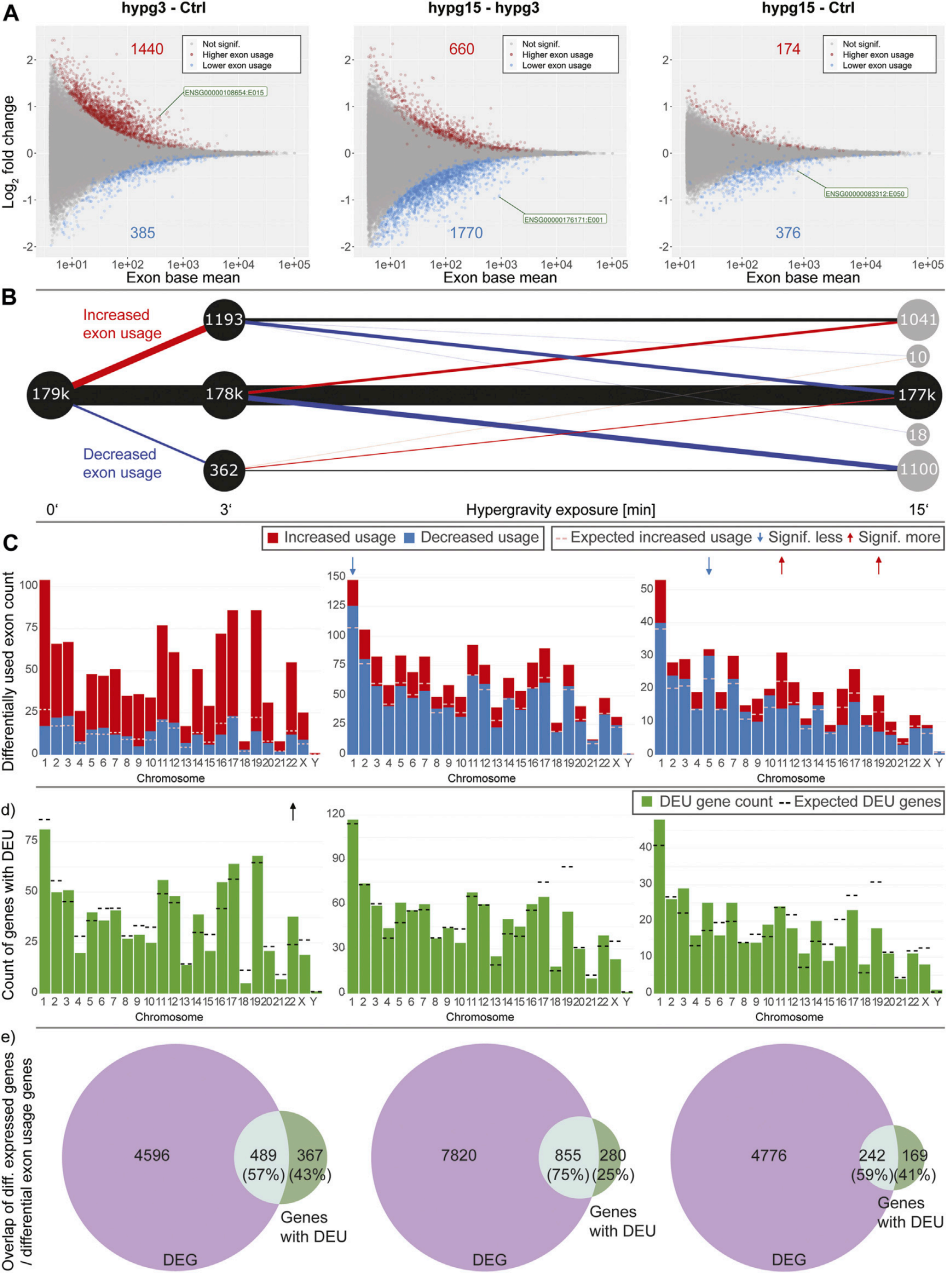
Splicing dynamics. **(A)** MA plot for fold changes of exons for all three comparisons in time. Exons that showed significantly increased or decreased usage are highlighted. The exon with the lowest *p* value is highlighted for each comparison. A skew towards upregulation and overall diminishing effect strength over time become visible. **(B)** Temporal coherence of differentially used exons. For exons that were called differentially used, the behavior between 3 and 15 min resp. after 15 min is shown. Only exons that have a non-NA false discovery rate (FDR) value for all three contrasts are shown, leading to smaller numbers of differentially used exons after 3 min. The same filter logic as for Figures 1C, 2A,B was used. The line width for non-significant exons has been scaled to 1/10th to assure visibility. **(C)** Distribution of exons with significant differential usage over all chromosomes for all three comparisons. The expected number of exons with increased usage per chromosome is indicated with a dashed line. If the deviation between the expectation and the actual number is significant, it is indicated with an arrow on top of the diagram, comparable to Figure 4. Exon usage tightly followed the expectation for most chromosomes, only for the later comparisons do deviations become evident. **(D)** Distribution of genes that showed significant differential exon usage over all chromosomes. Expected number of genes per chromosomes is indicated with a dashed line. **(E)** Overlap between genes that showed differential gene expression and genes that showed differential exon usage for the given comparison. A large fraction of genes simultaneously showed differential gene expression and differential exon usage, yet there is a sizeable fraction (25–43%) of genes that singularly displayed differential exon usage.

Gravitational force-dependent differential exon usage has been previously described to affect splicing in other organisms (Beisel et al., 2019). A full transcriptome splicing analysis was conducted to understand whether hypergravity-induced altered splicing was restricted to single genes or occurred in large parts of the genome. With stranded paired-end 75 bp reads, the RNA-Seq dataset from this study allowed robust identification of genes with differential exon usage (DEU), resulting from alternative splicing, alternative transcription start or end sites, or other forms of alternative isoform expression. Several differentially used exons could be identified for all three comparisons (Figure 4A), with a trend towards exons with significantly increased usage after 3 min (1440 versus 385 with significantly decreased usage). Between 3 and 15 min, exons with significantly decreased usage dominated. The overall effect diminished for the comparison hypg15-Ctrl, with a similar number of exons with significantly decreased usage as after 3 min and only 174 exons with increased usage. When looking at the temporal dynamics of exon usage, a rebound-like effect emerged (Figure 4B). This is not an artifact of the rebound pattern for overall expression since the utilized pipeline DEXSeq normalizes expression effects on the transcript level. Only half of the exons that were significantly increasingly used after 3 minutes were still increasingly used after 15 min, but an additional pool of exons that were initially not differentially used emerged. The same held true for exons with decreased usage (Figure 4B). The overall pattern therefore resembled a rebound effect. These significantly differentially used exons were distributed over all chromosomes with similar ratios of exons with increased versus decreased usage (Figure 4C). Only for hypg15-hypg3 and hypg15-Ctrl, a slight imbalance of the ratio of increased and decreased usage could be detected on chromosome 1 (hypg15-hypg3) and on chr5, chr11, and chr19 (hypg15-Ctrl). When shifting focus from the distribution of single exons towards entire genes that were significantly affected by differential exon usage, a pattern expected from overall gene distribution emerged (Figure 4D). Only chromosome 22 carried significantly more DEU genes than expected after 3 min. All other chromosomes approximately followed the expected distribution at all timepoints. Concludingly, differential exon usage acts on genes of all chromosomes equally. The distribution of increased versus decreased usage of exons was even more homogenous than the distribution of increased versus decreased differential gene expression (compare Figure 3).

Looking at the overlap between genes that were significantly differentially expressed (DEG) and those that showed significant differential exon usage (DEU) (Figure 4D), the number of genes with differential exon usage was small (489 + 367 = 856 after 3 min) compared to differential gene expression (4596 + 489 = 5085 after 3 min). Significant DEG and DEU genes only overlapped partly, but a major fraction of genes with DEU simultaneously were differentially expressed (Figure 4D, light green). Intriguingly, between 25 and 43% of genes were singularly affected by differential exon usage (Figure 4D, dark green). These formed a group of genes that transcriptionally reacted to altered gravity but did not appear in the pool of differentially expressed genes at these times. Therefore, differential exon usage could be a gravity-related effect that can act on genes independently of differential gene expression.

### Differential Exon Usage was Related to the Transcript Type

**FIGURE 5:**
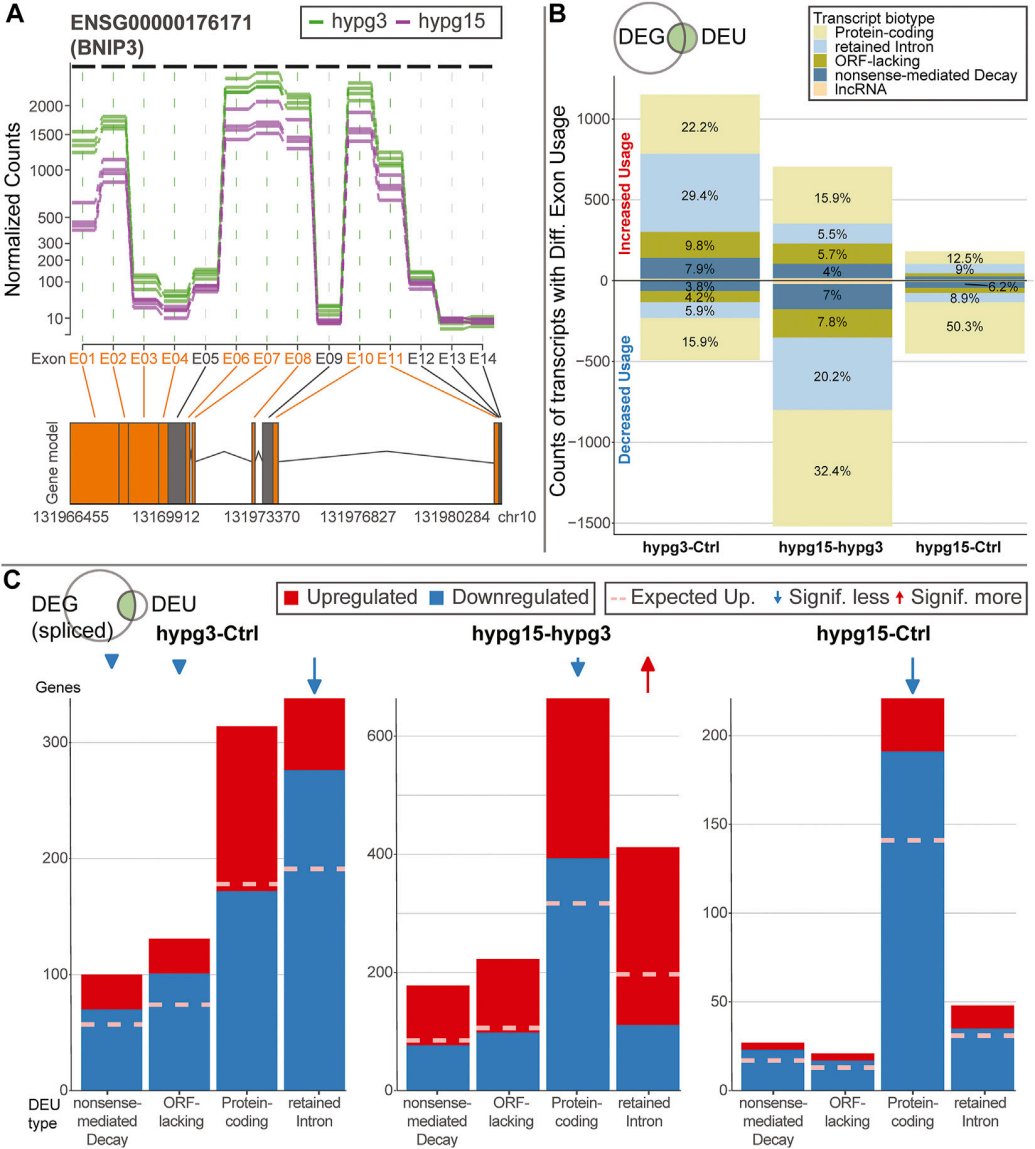
Characterization of effects of alternative splicing. **(A)** Example of differential exon usage for the gene BCL2 interacting protein 3 (BNIP3) that carries the exon (E01) with the strongest differential effect for the contrast hypg15-hypg3, annotated in Figure 4A. Normalized counts of all exons are displayed over the entire gene. Exons that were flagged as significantly differentially used are highlighted in orange on the axis label and in the schematic gene model (exons as boxes, introns as triangles). **(B)** Distribution of transcript biotypes for all differentially used exons for all three temporal comparisons. Alterations in protein-coding exons still lead to translatable transcripts; transcripts with exons flagged as retained introns, processed transcripts and nonsense-mediated decay lead to transcripts that are not translated into proteins or show decreased translation rates and are likely prone to early degradation. The overall diminishing effect strength after 15 min of hypergravity can be observed, additionally the fraction of DEU transcripts that are not protein-coding is prominent after 3 min and decreased after 15 min. **(C)** Plotting of alternatively spliced transcripts from genes that, in addition to differential exon usage (DEU), are differentially expressed genes (DEGs). For each transcript, up- (red) or downregulation (blue) of the host gene is shown. This is based on the spliced pool since per definition the unspliced pool should not be informative about alternative splicing (compare Supplementary Figure S10 for unspliced data). Data is split by transcript biotype on the x axis. lncRNA has been excluded due to the small number. The expected fraction of upregulated genes is indicated by a dashed line, based on the fraction of upregulated versus downregulated genes for all genes multiplied with the number of DEG-DEU overlaps for the specified exon biotype. Protein-coding transcripts constitute the dominant fraction after 15 min but not after 3 min. After 3 min, DEUs with retained introns are the dominant group and are additionally much more downregulated than expected, as opposed to protein-coding DEUs after 3 min.

To better understand the effects of alternative splicing, exons with significant differential usage were analyzed in more detail. A conceptual visualization of DEU is shown in Figure 5A. The exons of BCL2 interacting protein 3 (BNIP3), the gene with the strongest DEU in the contrast 15 versus 3 min, was analyzed. The read count per exon is shown for all samples from the two hypg3 and hypg15 groups. Several exons showed significant differences between the two conditions. Most prominently, exon E01 had an approximately 3-fold decrease in usage.

As a next step, the transcript isoforms that were affected by differential exon usage were categorized. Alternative splicing could for example affect one transcript isoform from a gene but not another isoform from the same gene. Consequently, for each exon of interest, all transcript isoforms that contained the specific exon were listed. These were annotated with their ensemble transcript biotype (categorization of transcript isoform), including protein-coding transcripts (those with an open reading frame), transcripts with retained introns (many could still code for proteins, but usually have complex regulatory behavior involved like localization, half-life etc.), transcripts lacking an open reading frame (ORF-lacking, general non-coding RNA except for lncRNA), nonsense-mediated decay (NMD, transcripts with premature stop codons that are prone to quick degradation), and lncRNA (Figure 5B). Overall, after 3 min, more transcripts showed increased than decreased exon usage. This was inverted for the comparison hypg15-hypg3, representing a rebound effect. Further, for the comparison hygp15-Ctrl, only a marginal effect appeared. A transcript can bear several exons, consequently these observations cannot easily be explained by the splicing dynamics demonstrated in Figure 4A. Strikingly, a skew between transcript biotype and increased or decreased usage of exons (upper or lower part of the plot) became evident: increased exon usage was disproportionally associated with transcripts with retained introns for hypg3-Ctrl. Also, ORF-lacking and NMD transcripts preferably had increased usage (upper part of the plot). However, protein-coding transcript isoforms were more evenly distributed with only a slight shift of 22.2 versus 15.9% towards increased exon usage. This effect inverted in the following 12 min: For hypg15-hypg3, transcripts with retained introns are shifted more towards significantly decreased exon usage. Therefore, the differential usage of exons at different points in time was related to the type of transcript the exon appears in: Increased usage of an exon initially appeared the most for transcript isoforms with retained introns. We could additionally quantitatively validate the trend towards intron retention after 3 min with the orthogonal package IRFinder (Supplementary Figure S9).

Intrigued by these findings, we wondered if these differential exon usage patterns were correlated with differential gene expression. We qualitatively analyzed if differential exon usage for a certain transcript biotype is associated with upregulation or downregulation of the corresponding gene. Therefore, transcripts that showed differential exon usage were matched with their harboring genes. These genes were then sorted by differential up- and downregulation. The datasets for all three comparisons were separated by the transcript biotypes from Figure 5B, protein-coding, retained intron, ORF-lacking, and NMD (Figure 5C). Only lncRNA was left out due to the small size of the group. The above was performed on the spliced dataset because by definition an unspliced transcript should not show alternative splicing and therefore act as negative control (for the unspliced data see Supplementary Figure S10). For the comparison hypg3-Ctrl, transcripts with retained introns were the largest group, as already expected from Figure 5B. The genes with splicing events on retained intron transcripts were significantly more downregulated than expected from the global distribution for hypg3-Ctrl (Fisher’s FDR-adjusted *p* value 2.9 × 10^−23^). The same held true for transcripts with nonsense-mediated decay (7.8 × 10^−3^) and ORF-lacking transcripts (2.0 × 10^−6^), but the effect was less pronounced. Between 3 and 15 min, the genes of retained intron transcripts were significantly upregulated (4.7 × 10^−18^). For hypg15-hypg3 and hypg15-Ctrl, the genes of protein-coding transcripts were skewed in up-/downregulation (1.7 × 10^−9^, 6.0 × 10^−14^). These specifically also appeared in the unspliced dataset (Supplementary Figure S10). Therefore, the effect on protein-coding was likely not caused by alternative splicing but by differential expression that happened pre-splicing.

We conclude that splicing events on intron retention and non-coding transcripts stem from genes that appear mostly downregulated after 3 min (Figure 5C). This held true only for genes on the spliced level. Therefore, we propose a link between alternative splicing of certain transcript biotypes and degradation of spliced transcripts.

### Differential Exon Usage Gene Downregulation was Associated With a Shift Towards Non-coding Transcripts

**FIGURE 6:**
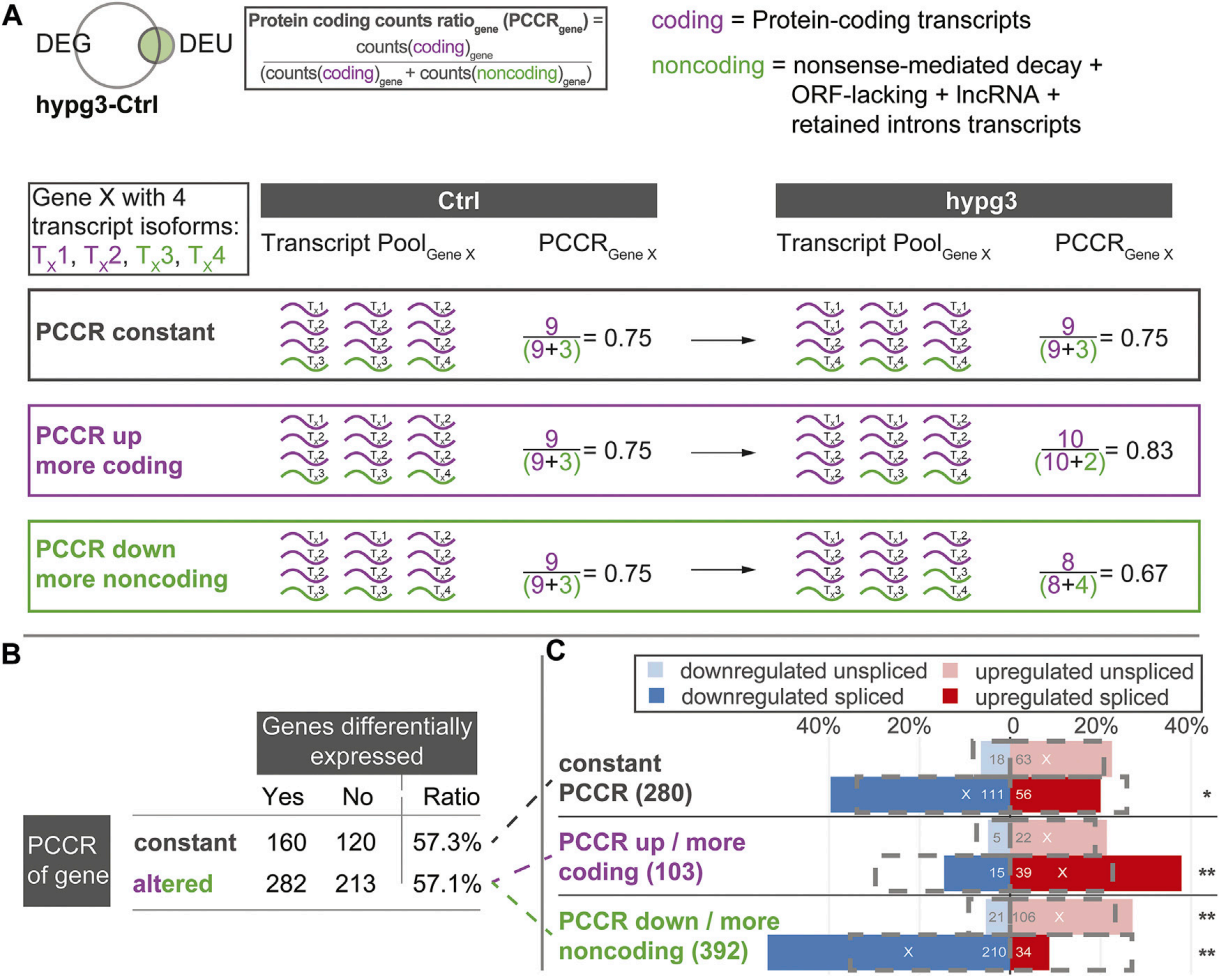
Protein coding counts ratio analysis of genes with differential exon usage. Quantitative analysis of the results from Figure 5C. Assessment if the transcript pool composition of genes changed between mostly coding transcripts and non-coding transcripts, and if this is associated with differential expression of these genes. **(A)** Definition of protein coding counts ratio (PCCR) as the fraction of RNA sequencing counts of coding transcripts over all counts of a given gene. Examples for a constant, increased, and decreased PCCR between Ctrl and hypg3 are given. **(B)** The fraction of differentially expressed genes was analyzed for genes where the PCCR was altered (up and down) and for those with unaltered PCCR. **(C)** Split between genes with constant PCCR, increased PCCR, and decreased PCCR. For each subset of genes, the number of genes that is significantly upregulated or downregulated in the spliced and unspliced fraction is indicated. The grey boxes indicate the expected distribution from the overall DEG datasets. Significant deviations from the expectation based on a Fisher exact test are indicated (** for *p* < 0.01, * for *p* < 0.05). An “X” indicates the mean direction of differential gene expression. An extended analysis can be found in Supplementary Figure S11.

Encouraged by the previous findings we wanted to further characterize the effects of alternative splicing on the composition of the transcript pool. We wanted to identify the fate of initially protein-coding transcripts, when different exons, like shown in Figure 5A, are incorporated upon altered gravity exposure. For example, if a protein-coding transcript is alternatively spliced in hypergravity and thereby loses an exon, does it become inactive or is it an alternative protein-coding splice isoform? The latter would count as splicing event, but it would not lead to a decrease in the number of protein-coding transcripts allocated to a certain gene. To obtain a measure of such effects, we defined the protein coding counts ratio (PCCR) (Figure 6A): For each gene, the number of transcripts were counted and divided into coding transcripts and “noncoding” transcripts. the group “noncoding” transcripts included the categories of transcript isoforms that are not protein-coding, i.e., NMD, ORF-lacking, containing retained introns, and lncRNA.

We analyzed the transcript distribution for DEU genes (compare Figure 4D) after 3 min of hypergravity (for other comparisons, see Supplementary Figure S11). For each DEU gene shown in Figure 4D, we determined the number of transcripts. The transcripts were then separated into protein-coding transcripts and noncoding transcripts and the PCCR (ratio between counts for coding transcripts versus all transcripts for a certain gene) was calculated (see formula in Figure 6A). If the PCCR was significantly altered after 3 min of hypergravity exposure, the corresponding gene was called “PCCR up/down”. We then further analyzed how many of these genes were additionally differentially expressed after 3 min (Figure 6B). At first sight, constant and altered PCCR showed the same amount of differentially expressed genes of about 57%.

We further analyzed the PCCR up/down genes and individually investigated those with a significant shift towards coding transcripts (PCCR up) and those with a significant shift towards noncoding transcripts (PCCR down) (illustrated by dashed lines between Figure 6B and Figure 6C). Additionally, we analyzed how many of these PCCR up/down genes were up- or downregulated in the spliced and unspliced transcript pools, respectively (Figure 6C).

Constant PCCR had a non-significant skew towards upregulation in the unspliced and a slightly significant (*) skew towards downregulation in the spliced transcript dataset. For genes with transcript compositions shifted towards coding (PCCR up), both spliced (significant) and unspliced (not significant) were shifted towards upregulation. Yet, the absolute number of such genes was around ×4 smaller (103) than of those with PCCR down (392). Genes with a shift towards noncoding transcripts (PCCR down) also showed a skew towards upregulation on the unspliced level. Strikingly, these genes with PCCR down were significantly (**) shifted towards downregulation on the spliced level.

These surprising findings highlight that the transcripts of genes undergoing alternative splicing are not in a steady state after 3 min of hypergravity (Figure 5). A dominant fraction belongs to transcript isoforms with retained introns (38.3% of DEU-DEG transcripts), for which the corresponding genes were mostly downregulated (Figures 5B,C). For genes with shifted transcript pool from protein-coding transcripts towards non-coding transcripts, a heavy skew towards downregulation could be observed in gene expression (Figure 6C, PCCR down). Surprisingly, this was accompanied by upregulation in the unspliced pool, suggesting that this subset of DEU genes showed increased transcription after 3 min.

### Downregulation Caused by a Shift Towards Noncoding Transcripts is Associated With Genes With Nucleolar Function

**FIGURE 7:**
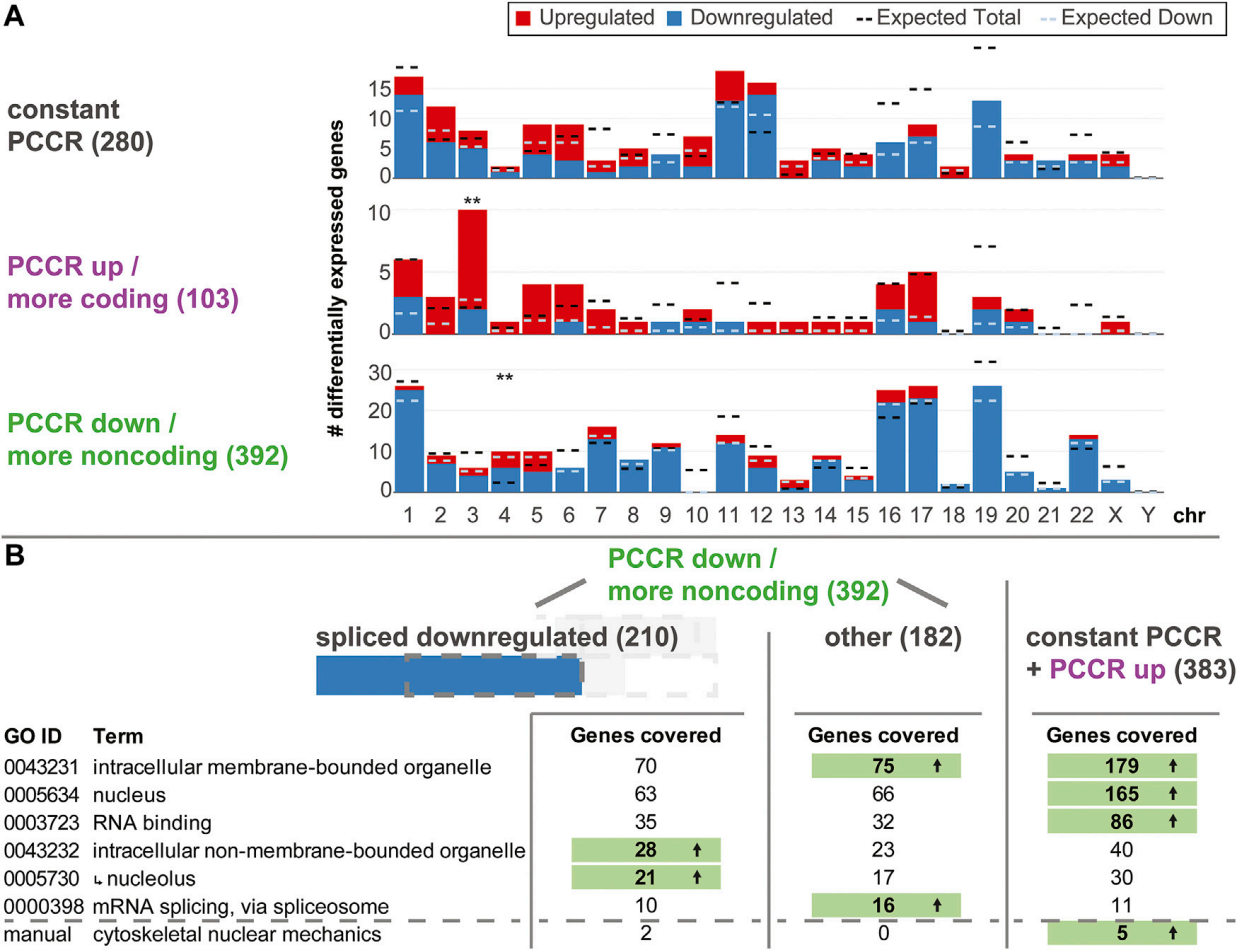
Functional analysis of genes with constant, increased, and decreased PCCR. **(A)** Number of differentially upregulated (red) and downregulated (blue) genes per chromosome (horizontal axis) for genes with constant PCCR, increased PCCR, and decreased PCCR. The expected number of total DEGs per chromosome (based on the fraction of differentially expressed genes for all genes and the number of detected genes per chromosome and assuming a uniform probability of differential expression) is shown as a black dashed line, the expected number of downregulated genes out of the set of up- and downregulated genes is shown as a dashed light blue line. Chromosomes with significantly more or less differentially expressed genes are indicated with black stars, those with a significant deviation in the distribution of up-versus downregulated genes with blue stars (FDR <0.05 *, FDR <0.01 **). **(B)** Gene Ontology (GO) enrichment analysis of genes with decreased PCCR that are downregulated in the spliced dataset (left), all remaining genes with decreased PCCR (middle), and those with constant or increased PCCR (right). Only gene Ontology sets covering a large fraction of genes from the groups are listed. Further, a manually curated gene set was analyzed, containing factors involved in the cytoskeletal nuclear mechanical axis. If the number of covered genes significantly (FDR <0.05) exceeds the statistical expectation, it is highlighted in green with an arrow. The *nucleolus* GO set is a subset of the *intracellular non-membrane-bounded organelle* set, as indicated by an arrow.

Driven by the findings that altered PCCR is highly associated with differential levels of spliced transcripts, we wondered what the physiological implications of such a process could be. First, we asked if such effects were specific to certain chromosomes or chromosome bands (Figure 7A). For all three types of PCCR (constant, increased, decreased), the number of up- and downregulated genes was plotted per chromosome, similar as in Figure 3. Effects occurred on all chromosomes except chrY. Only chr3 for genes with increased PCCR and chr4 for genes with decreased PCCR showed significantly more differentially expressed genes, however deviations were in the single digit range. Therefore, effects on PCCR-associated differential transcript levels were not chromosome-specific.

Next, we asked if downregulated genes with decreased PCCR could play any functional role. An enrichment analysis for gene ontology sets was performed on the group of genes with decreased PCCR that appeared downregulated on the spliced level, all other genes with decreased PCCR, and genes with either constant or increased PCCR (Figure 7B). For sets coving a large fraction of genes, we checked if any contained more target genes than expected by random drawing. Results were diametrically different for the different subgroups: For decreased PCCR genes that were not downregulated on the spliced level (Figure 7B, middle), and for constant and increased PCCR (Figure 7B, right), *intracellular membrane-bounded organelles* (GO:0043231) contained more genes than expected. For constant and increased PCCR, additionally *nucleus* and *RNA binding* contained more genes than expected. *mRNA splicing*, *via spliceosome* was enriched in the group of genes with decreased PCCR that are not downregulated on the spliced level. For decreased PCCR genes that were downregulated on the spliced level, however (Figure 7B, left), none of these groups was enriched. The set of *intracellular non-membrane bounded organelle* (GO:0043232) and its subset *nucleolus* (GO:0005730) were highly enriched with *p* values <0.01 for both sets. More than 10% of the genes that showed PCCR decline-associated downregulation were contained in these sets.

Further, a manually curated cytoskeletal nuclear mechanical axis set was analyzed. It included lamin A/B/C genes LMNA and LMNB, the two cytoskeletal actin genes, ACTB and ACTG1, the two tubulin isoforms TUBA1A and TUBB; from the LINC complex the relevant nesprins SYNE1-SYNE4, SUN1 and SUN2, and Emerin; and from the nuclear pore complex (NPC) all nucleoporins NUP, POM121, Tpr, NLP1, RAE1, and further NPC-associated genes IPO, TAP, Ran, RCC1, Hsc70, VIM, UBC9, and SENP2, summing to 55 genes in total. In the group of downregulated genes with decreased PCCR, Actin B and the nuclear pore complex gene RAE1 were included. The other cytoskeletal isoform of Actin, G1, was covered in the neutral PCCR fraction (affected by alternative splicing, but no shift in coding versus noncoding distribution). Further, Tubulin A1B, nuclear pore complex (NPC) gene NUP98 and the NPC-associated genes RANBP1 and POM121C were part of the neutral PCCR fraction. Here, the set is significantly overrepresented in the constant PCCR fraction. In the comparison hypg15-Ctrl, ACTG1 and TUBA1B (instead of 1A) are still affected by alternative splicing.

### Potential Regulators of Transcriptional Rebound Effects: RNA Processing Factors, Transcription Factors, Translation Machinery, and Cellular Compartments

The previously described transcriptional rebound effect (compare Figure 1 for differential expression rebound, compare Figure 4 for differential exon usage rebound) requires rapid cellular reactions to be able to invert the transcriptional response on a time scale of minutes. To find further potential mediators of such a response apart from nucleolus-associated translational inhibition, several cellular factors that are known to modulate transcriptional responses were analyzed. If a pattern of a factor aligned with observed differentially expressed genes, this could be a potential contributor to the observed rebound effect.

**FIGURE 8:**
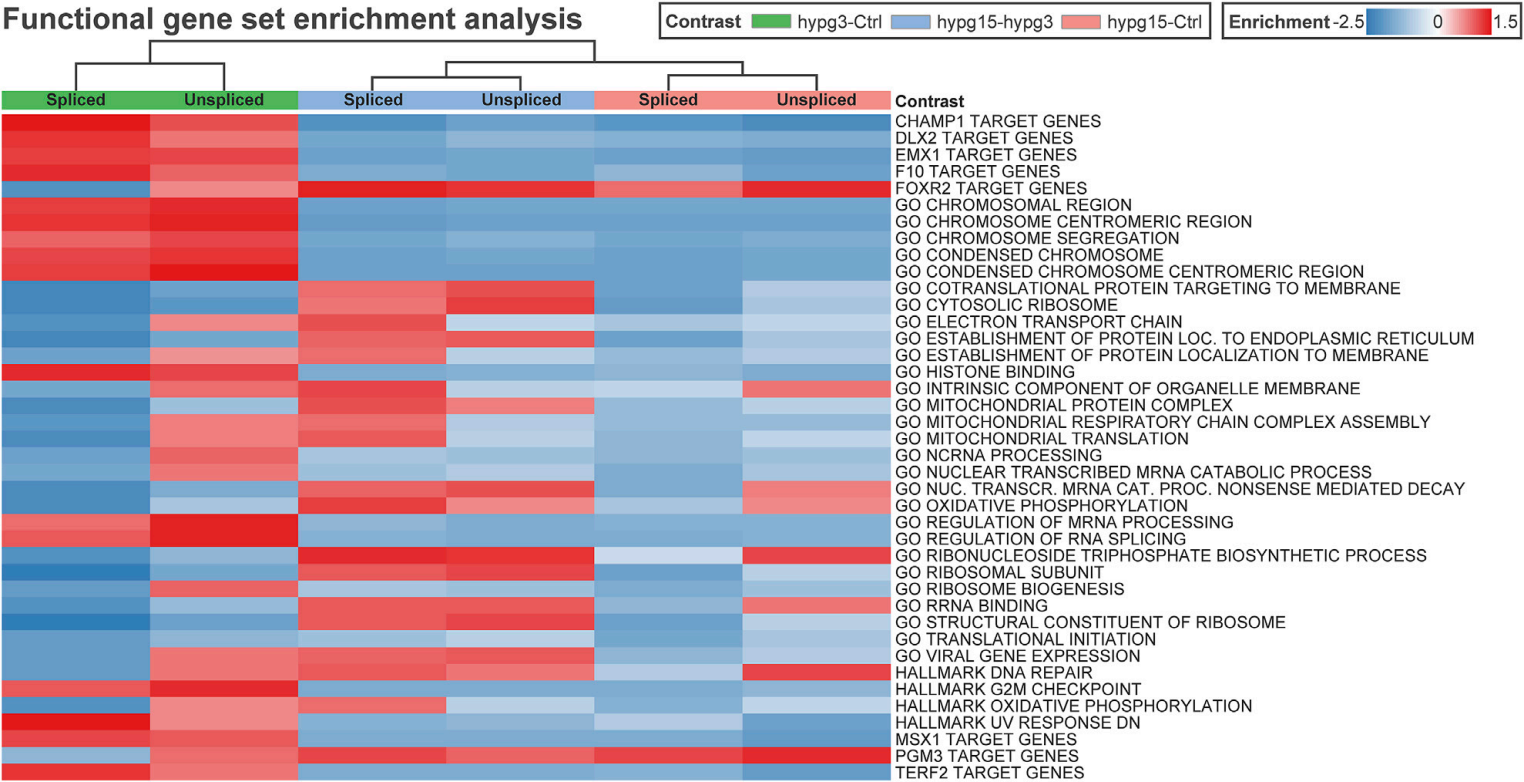
Correlating regulatory effects with differential gene expression. Functional gene set enrichment analysis for differential gene expression for all three comparisons. The analysis was conducted separately for unspliced and for spliced transcripts. Selected MSigDB sets were analyzed, including GO pathways, transcription factor targets, hallmark genes, etc. Most gene sets show a rebound effect with either upregulation after 3 min and consecutive downregulation between 3 and 15 min or the other way around. Only a few sets show consistent enrichment for both spliced and unspliced data, including GO *translational initiation* which is consistently downregulated.

To get an impression if transcription factors or other cellular pathways could be acting on the pool of transcripts, a gene set enrichment analysis was performed on the spliced/unspliced transcript pools of all three comparisons (Figure 8). Clustering led to grouping of data subsets by comparisons, rather than splicing states. Several transcription factor target sets emerged, including DLX2, EMX1, FOXR2, MSX1, and PGM3. Except for FOXR2 and PGM3 target genes, all showed significant enrichment after 3 min and the opposite effect in the other comparisons. Further, several Gene Ontology sets emerged that were associated with chromatin structure or histone binding, mitochondrial processes, RNA processing such as splicing, and translation-associated pathways like translation initiation (consistently downregulated) and ribosome-associated factors.

Stress granules and P bodies are cellular compartments involved in scavenging RNA in stressful settings. Stress granules form due to different types of cellular stress, bind certain transcripts, and mark them for degradation to prevent excessive translation (Decker and Parker, 2012; Matheny et al., 2019). P bodies are constantly present but have a similar function in RNA homeostasis by binding and degrading RNA molecules. Based on a correlation analysis, the potential of these structures to buffer the initial transcript upregulation was assessed (Supplementary Figure S12). For the hypg3-Ctrl comparison, 2x more genes with stress granules/P bodies affinity were upregulated and much less downregulated, than expected. This renders them potential targets for later investigations on regulators of the transcriptional rebound effect.

## Discussion

Recently, we discovered that the transcriptome responds rapidly and in a complex manner to altered gravity (Thiel et al., 2017b; Thiel et al., 2018; Vahlensieck et al., 2021a; Vahlensieck et al., 2021b). Fundamental alterations in the cellular transcription pool as a reaction to short-term microgravity and hypergravity between 20 s and 5 min appear in different immune cell types and include 1000s of genes (Thiel et al., 2017b; Thiel et al., 2017c; Thiel et al., 2018). In these studies, transcriptional reactions were compared within the same cell type for different points in time on two different platforms. We proposed a rapid transcriptional adaption reaction based on the findings that transcripts that are differentially expressed after 20 s of hypergravity/microgravity are no longer differentially expressed in the same direction after 75 s of hypergravity/5 min of microgravity. Using a cross-experiment approach, we recently could further demonstrate that exemplary genes and also gene sets show inverse behavior after 15 min of hypergravity, compared to these initial effects (Vahlensieck et al., 2021a). However, cross-platform effects and different transcriptomic methods might introduce false positive hits and do not capture all significant differences (Vahlensieck et al., 2021a). Therefore, true time course-related effects that are influenced by exposure time to altered gravity cannot be fully separated from potential artifacts.

Consequently, this study assessed the temporal effects of hypergravity based on data points that were acquired under similar and highly standardized conditions. The first is a 3 min time point in the earliest known phase of transcriptional reactions towards stress (Mahat et al., 2016), within the temporal range (20 s, 75 s, 5 min) of altered gravity platforms used by our group (Thiel et al., 2017b). The second sample group was taken at 15 min of hypergravity, therefore towards a potential steady state (as described in (Vahlensieck et al., 2021a)). We were able to identify underlying patterns with temporal differences in the transcriptional response to short term hypergravity.

One central aspect we discovered for Jurkat T cells is the transcriptional rebound effect, where gene expression is initially altered in one direction but then inverts with transcript levels of differentially expressed genes either no longer detectable as altered or changed in the opposite direction (Figure 1). Concomitantly, a pool of genes with initially unchanged transcript levels emerged as differentially expressed. In the previously described studies, transcripts that emerged as differentially expressed after 20 s were mostly no longer differentially expressed after 5 min of altered gravity, and new differentially expressed genes appeared (Thiel et al., 2017b). The previous findings can be explained by a rapid rebound mechanism. The rapid homeostatic response of the transcriptome could be seen in the context that life evolved under the permanent influence of constant gravity (Morey-Holton, 2003).

Further, we were able to separate differential gene expression into effects on the spliced and the unspliced level, enabling us to separate transcriptional effects that act only on spliced transcripts, unspliced transcripts, or both (Figure 2). Most prominently, initial effects after 3 min were very different between the spliced and the unspliced level with regards to up- and downregulation. While the magnitude of initial upregulation was comparable between spliced and unspliced levels, immediate downregulation of gene expression almost exclusively acted on the spliced pool, with delayed downregulation in the unspliced pool. Therefore, the effect that downregulates genes after 3 min must act only on transcripts after splicing but not prior to splicing, a post-transcriptional effect [compare (Gaidatzis et al., 2015)]. This is in line with initial post-transcriptional downregulation [reviewed in (Day and Tuite, 1998)], for example by active degradation, but not with a lowered transcription rate of these genes as initial cause. Between 3 and 15 min, unspliced downregulation was strongly increased, supporting a mechanism that directly acts on transcription. The latter could be related to general stress response mechanisms in which overall transcription is strongly decreased. Indeed, this has been described for thousands of genes within 10 min of exposure to heat or celastrol (Duarte et al., 2016; Mahat et al., 2016; Vihervaara et al., 2017; Vihervaara et al., 2018), within the same timeframe and a similar magnitude to our observations. Decreased transcription of certain genes as a general stress response could therefore be a secondary effect but not directly and initially connected to gravity-sensing.

Generally, we could reconfirm the large and genetically global scale of differential gene expression, as already postulated in previous work (Thiel et al., 2017b). The homogenous distribution of differential gene expression over the chromosomes and the specific distribution of up- and downregulated genes in chromosome bands allows the formulation of hypotheses regarding underlying mechanisms (Figure 3). We found that transcriptome response to altered gravitational force did not only affect single chromosomal regions or singular sets of genes (also compare gene set effects) but act on the entire genome with comparable effect strength, whereby the specific pattern of up- and downregulation on the level of chromosomes/chromosome bands supports a mechanism that codes for the location in the genome. Initially, the number of affected genes per chromosome matched expectation from random drawing. For the hypg15-hypg3 and even more for hypg15-Ctrl comparisons, this is no longer true. This could be an indication of transcriptional feedback loops, where gene expression of certain genes is coupled to others, thereby either activating other genes or moving back to a steady state, which could progressively disturb the DEG distribution of all chromosomes (Moss Bendtsen et al., 2015). The detected hypergravity effects are in line with the previously proposed gravitational sensing mechanism based on mechano-genetic coupling effects in chromatin architecture (Vahlensieck et al., 2021b): Here, it could be shown that gravi-reactive chromosomal regions are conserved between experiments at different time scales and a direct link to conformational changes of the chromatin could be demonstrated. This would explain why the entire genome was affected in our study, but different regions react differently. Interestingly, chromosome 19 had the strongest skew for absolute number of DEGs after 3 min and the strongest relative skew at all points in time. As previously reported, chromosome 19 is among the most affected by conformational effects of altered gravity on Jurkat T cell chromatin (Vahlensieck et al., 2021b).

The analysis of differential exon usage and of the protein coding counts ratio (PCCR) adds a puzzling novel perspective on transcriptional dynamics under hypergravity (Figures 5, 6): Initial effects after 3 min involved primarily an increased usage of exons in noncoding transcripts, accompanied by a qualitative shift towards noncoding transcripts (Figure 5). We further performed a quantitative analysis and introduced PCCR (Figure 6). For many genes with differential exon usage (DEU genes), downregulation on the spliced level was associated with a shift from protein-coding transcripts to noncoding/intron-retaining transcripts (decreased PCCR). This did not affect the unspliced level and therefore is a post-transcriptional effect. Many of these noncoding transcripts are prone to quicker degradation *via* several pathways, above all nonsense-mediated decay transcripts (Kurosaki et al., 2019). Many intron-retaining transcripts have a preference towards degradation and reduced translation (Wong et al., 2016), and a lacking open reading frame correlates with quick decay (Decourty et al., 2014). On the other hand, one must consider that the largest fraction of RNA that has been generated by RNA Polymerase II in mammalian cells remains in the nucleus and is consecutively degraded in there (Jackson et al., 2000). Generally, the localization of mRNA is a tightly regulated process with many associated regulatory factors (Parton et al., 2014). Therefore, two explanations emerge that cannot be discriminated: 1) A shift in splicing dynamics resulting in more noncoding transcripts leads to downregulation of such genes for spliced transcripts, while unspliced transcripts remain unaffected. Consequently, downregulation of genes with lowered PCCR can be explained by noncoding transcript degradation and therefore could promote the transcriptional rebound effect: Initially, genes are either overexpressed or constant in the unspliced fraction. Next, they get spliced differentially, shifting the PCCR. Then, these newly appearing noncoding transcripts get degraded (Decourty et al., 2014; Smith and Baker, 2015; Wong et al., 2016; Kurosaki et al., 2019), as visible in the spliced pool. 2) A gravity-induced inhibition of the export of spliced protein-coding mRNA exposes them to nuclear mRNA degradation. For protein-coding mRNA, the nucleus often poses a much higher risk of rapid degradation than the cytosol (Schmid and Jensen, 2018). This would reduce the fraction of mRNA labeled for cytosol export but not the fraction that would not leave the nucleus, anyways. Noncoding transcripts mostly have a preference for nuclear localization (Statello et al., 2021). Thereby, the overall amount of mRNA of a certain gene is reduced, leading to downregulation, whilst shifting the PCCR since the additional degradation acts on protein-coding mRNA that would normally be exported. This mechanism would fit the findings that the nuclear pore complex, the gatekeeper of cytoplasm-nucleus transition, changes its conformation and thereby its export capacity upon altered membrane tension (Zimmerli et al., 2021) and our findings that the nuclear conformation and therefore also likely membrane tension is affected by differential gravity (Vahlensieck et al., 2021b). For both mechanisms, degradation would initially only occur in the spliced fraction and upregulation would be skewed towards the unspliced fraction, as visible in Figure 2A. Later, as differential splicing or export inhibition diminishes (Figure 5A), the skew between upregulation and downregulation for the spliced and unspliced fraction gradually disappears (Figure 2C, hypg15-Ctrl). In this later phase, pre-transcriptional inhibition would explain the observations best. It remains unclear if the potential mechanisms only affect a small fraction of genes, as observed here, or is the main mechanism behind initially downregulated genes. The sequencing parameters, with 75 bp paired-end reads, allowed a solid quantification of alternative splicing for some genes, but increased read length would drastically enhance detection power, allowing detection of PCCR shift for many more genes (Chhangawala et al., 2015). Therefore, the current dataset only allowed robust isoform analysis of a fraction of genes.

Alternative splicing in altered gravity (Figure 4) has not been systematically described in mammalian cells previously. Interestingly, we detected an increased retention of exons in the first 3 min that would normally be spliced out, not only in the particular cases where the PCCR is affected. This could be the caused by an inhibition of splicing in hypergravity, e.g., *via* gravity-sensitive epigenetic modifications (Tauber et al., 2015) such as histone methylation that regulates splicing (Luco et al., 2010; Ringrose, 2010), but also because the splicing system is challenged by the increased transcription rates, to which exon selection is very sensitive (de la Mata et al., 2011; Dujardin et al., 2014; Ramanouskaya and Grinev, 2017). Intron retention has been described as a global effect in 27% of uninduced multi-intron genes in mouse fibroblasts during heat stress, caused by hindrance of post-transcriptional splicing in particular (Shalgi et al., 2014). These transcripts are retained in the nucleus and remain untranslated. Such a stress response mechanism could also apply in the present case of gravity-related cell stress. In *Arabidopsis* exposed to microgravity on the International Space Station, global effects of alternative splicing have been described to lead to transcript isoforms that cannot be detected on the ground (Beisel et al., 2019). Splicing effects of altered gravity could be of relevance for immune cell function: It has been described that T cells undergo alternative splicing during their activation and that immune cells in general rely for their abilities on alternative splicing (Ip et al., 2007; Martinez et al., 2012; Martinez and Lynch, 2013). An effect that disturbs alternative splicing patterns could therefore provide another layer of explanation for observed immune deficiencies during spaceflight (Choukèr and Ullrich, 2016).

Considering the discrepancy between spliced and unspliced transcripts after 3 min that becomes significantly smaller after 15 min (compare Figure 2), and considering that most of the differential exon usage effects are no longer present after 15 min (compare Figures 4–6), the cell behaves fundamentally differently for the comparison hypg3-Ctrl than for hypg15-Ctrl. The very first initial effects indicate a status far away from a steady state, whereas cells potentially approach a new steady state equilibrium during the homeostatic response. This has been reported for cellular systems that are exposed to rapid-onset, permanent stimuli, including the response to galactose in yeast (Braun and Brenner, 2004) and to heat shock in bacteria (Kim et al., 2020), *Drosophila* (Duarte et al., 2016), and murine cells (Mahat et al., 2016), and seems to be a conserved feature of cellular stress response (López-Maury et al., 2008). The murine heat shock study differentiated between quickly induced genes which were upregulated after 2.5 or 12 min and were mostly downregulated after 60 min, and genes with later induction that appear upregulated after 60 min, corresponding to the rebound effect described here. Interestingly, the number of differentially expressed genes was on the same order of magnitude as described here with ∼1500 upregulated and ∼8000 downregulated genes after 60 min.

The heat shock study in bacteria also separated the transcriptional response into an early response stage (2–10 min), a mid-term response stage (30 min-2 h), and a late response stage (4–48 h) with distinct groups of genes. This suggests a rapid dynamic response in the early stage, subsequent adaption towards a steady state in the mid-term stage and long-term thermotolerance at a new steady state in the late response stage. Such overshoot and subsequent novel steady state dynamics have been described to accelerate cellular adaption periods to new environments (López-Maury et al., 2008).

The large number of differentially expressed genes, taken together with the relatively small fold changes below factor 2 for most genes (compare Figure 1B), could also be regarded in the light of an early-stage response. After 3 min, the cell is in a non-steady-state condition: Considering an average mRNA half-life time in human cells of several minutes and hours (Yang et al., 2003; Chan et al., 2018), the majority of mRNA for a specific gene originated from pre-treatment transcription. Additionally, the used polyA library allows quantification of fully transcribed (pre)-mRNA, thereby underestimating the RNA molecules that did not finish transcribing within the time frames. This currently limits the analysis to transcripts that already host readily assembled transcription complexes and were transcribing, and to genes short enough to be fully transcribed within 3 min. Considering the relatively slow RNA Pol 2 elongation speed of on average 2 Kb-6Kb/sec (Fuchs et al., 2014) and the behavior of extended pause intervals of RNA Pol 2 (Darzacq et al., 2007), transcription initiation and full transcription of most genes [median length 27 kb (Cunningham et al., 2022)] will not be possible within the first 3 min. It therefore seems likely that initial up-regulatory changes mostly result from increased elongation rate. Tight coupling of RNA elongation with the epigenetic state of chromatin like histone modifications (Veloso et al., 2014; Jonkers and Lis, 2015) and with 3D conformation (Papantonis and Cook, 2010; Heinz et al., 2018) would allow to associate our previous results on differential effects of altered gravity on histone modifications (Tauber et al., 2013) and on chromosome conformation (Vahlensieck et al., 2021b).

In a pathway analysis (Figure 8), multiple GO pathways emerged that are associated with chromatin structure, histone binding, mitochondrial processes, RNA processing such as splicing, and further translation-associated pathways like (consistently downregulated) translation initiation and ribosome-associated factors, potentially involved in cellular adaptations to initial hypergravity-associated transcriptional stress. The GO gene sets *intracellular non-membrane-bounded organelles* and in particular *nucleolus* were significantly overrepresented for the downregulated genes (Figure 7B). As the production site of ribosomes, regulation of nucleolar proteins including change in size of the nucleolus and ribosome biogenesis factors can directly affect translational efficiency (Weeks et al., 2019), for example during heat stress (Cherkasov et al., 2015). Therefore, the PCCR-associated downregulation of nucleolar genes could promote long-term decline of translation as a cellular stress adaption. Further, as a central response hub to stresses, such as hypoxia, pH changes, redox stress, and DNA damage (Weeks et al., 2019), the nucleolus could be a central player in the gravitational stress response. Initial evidence supports these findings: Cellular ribosome protein content is significantly reduced in neonatal rat cardiomyocytes after 120 h of simulated microgravity (Feger et al., 2016). In *Arabidopsis*, after 24 h of simulated microgravity, ribosome biogenesis indicators are significantly reduced and an increased number of nucleoli were inactive (Kamal et al., 2018). Of course, correlation between gene expression and protein content is generally modest, and not always given (Khan et al., 2013; Battle et al., 2015). Often used as a proxy for protein content, transcriptomics studies always bear the problem that it is unclear if the transcription of a certain gene propagates to an increased concentration of the target protein (Buccitelli and Selbach, 2020). In particular, 1% of mammalian genes show a high transcription but a low translation rate (Hausser et al., 2019), likely to allow rapid translation upon stimulus instead of waiting for novel transcription to significantly raise mRNA concentrations (Buccitelli and Selbach, 2020). Generally, transcription is considered an energetically cheap mechanism while translation is considered to drain resources and have a longer legacy in the cell, given the longer half-life of proteins compared to RNA. Therefore, cells stabilize their protein levels instead of mRNA concentrations, an effect conserved throughout kingdoms (Schwanhäusser et al., 2011; Lalanne et al., 2018). For example, only in 1/3 of cases, protein concentrations significantly differ between primate species where mRNA content is significantly different (Khan et al., 2013). One prominent method of limiting translation to save energy under stress conditions both in bacterial and mammalian cells is translational hibernating by converting active polysomes into pairs of ribosomes that do not longer translate (Krokowski et al., 2011), which can be rapidly converted back into active ribosomes upon stress release. Further there is an active pausing response where ribosomes stall at initiation codons as a coordinated stress response (Jobava et al., 2021). This gives a hint to understanding the pronounced transcriptomics effects, including differential exon usage, in the light of cellular stability and a tendency towards a new transcriptional steady state: as long as transcription is highly impeded, cells are able to limit dangerous effects on the protein pool.

**FIGURE 9:**
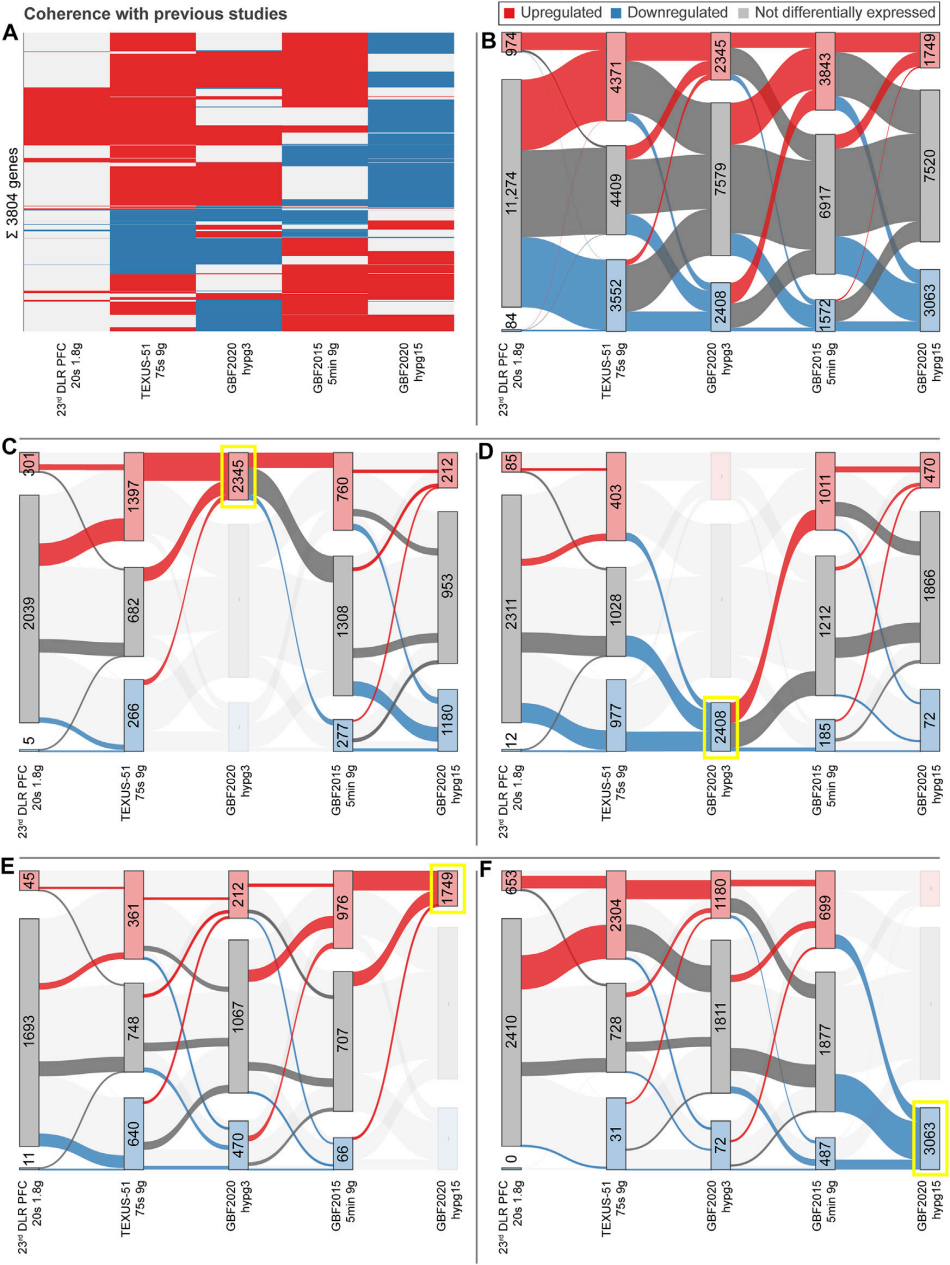
Coherence of transcriptional dynamics with other hypergravity studies at different timepoints. Hypergravity comparisons from previous studies have been added to the two comparisons hypg3- Ctrl and hypg15-Ctrl, including 20 s of 1.8xg hypergravity from the 23rd DLR Parabolic Flight Campaign (PFC), 75 s of on average 9xg hypergravity during the TEXUS-51 campaign, and 5 min of 9xg hypergravity from the 2015 ground-based facilities campaign (GBF 2015). Data sets are filtered for genes that could be detected in all data sets. Data sets are ordered by exposure time. **(A)** Clustered heat map of all genes that are differentially expressed in at least 3/5 of the data sets, a total of 3805 genes. Significantly (FDR <0.05) upregulated genes are indicated in red, downregulated in blue, not significantly differentially expressed in grey. **(B**–**F)** Sankey flowchart of gene expression behavior over time. For each data set, corresponding to a point in time, the number of significantly (FDR <0.05) upregulated (red), not differentially expressed (grey), and downregulated (blue) genes is given. Box heights are proportional to the number of genes. For each category at each timepoint, the number of genes that consecutively become upregulated, downregulated, or not differentially expressed at the next timepoint is indicated by colored connectors of different sizes. This is either performed for all genes **(B)** or highlighting the behavior of genes that are upregulated or downregulated at a certain point in time, including upregulated after 3 min **(C)**, downregulated after 3 min **(D)**, upregulated after 15 min **(E)**, and downregulated after 15 min **(F)**.

To sum up, this study attempted to provide a hypothesis for early transcriptional effects and their homeostatic regulation caused by altered gravity in this very specific experimental setting. While extensive and highly standardized multi-timepoint studies from one campaign would be preferable, comparison of the existing five short-term hypergravity time points already allow for a robust hypothesis test (Thiel et al., 2017b; Thiel et al., 2017c; Vahlensieck et al., 2021a). These were all recorded on microarray technology, therefore detailed studies of unspliced versus spliced transcripts and calculations of PCCR effects are not possible. Nevertheless, if the expression patterns of the five data sets resemble different time points of the same shared process, the transcriptional rebound effect should coherently emerge when comparing all of them (Figure 9). Therefore, we assessed the data sets for coherence, i.e., if the direction of differential expression at one point in time compares to the previous point in time, e.g., if a number of genes were upregulated, are still more genes upregulated than expected from random drawing at the next point in time. We compared the two internal comparisons **hypg3**-Ctrl and **hypg15**-Ctrl from this GBF2020 campaign, a 20 s 1.8xg versus 1xg inflight comparison from the 23rd DLR Parabolic Flight Campaign (Thiel et al., 2017b), a 75 s ∼9xg versus 1xg ground comparison from the TEXUS-51 mission (Thiel et al., 2017b), and a 5 min 9xg-Ctrl GBF comparison from the GBF2015 mission (Thiel et al., 2017c). Comparative analyses were performed despite differences in analytical methods (microarray vs. RNA Seq) and experimental conditions (real flight experiments versus lab study), because particularly robust effects should be confirmed by independent experimental procedures, thus overcoming the fundamental disadvantage inherent in high standardization: The difficulty of interpreting data collected in a highly artificial system in the context of biological reality.

On a qualitative basis (Figure 9A), coherence is given: hypg3, 75 s, and 20 s show the same direction of gene expression for the majority of genes, with a much lower number of DEGs for the 20 s data set, probably because of the short time. Hypg15 shows the opposite direction of regulation for most genes. Only 5 min shows characteristics of both the early response and late response. This time point could be close to a (hypothetical) inversion point of the transcriptional rebound. Further, when comparing the direction of regulation quantitatively (Figure 9B), coherence is given between the first three data sets (early response). Upregulated genes stay upregulated or do not count as differentially expressed at 20 s, 75 s, 3 min, and partly at 5 min. However, between 75 s and 3 min and between 3 and 5 min, large fractions are not differentially expressed that were upregulated previously and *vice versa*. Downregulated genes remain downregulated to a lesser extent and mostly become not differentially expressed again between 75 s and hypg3. Flips between up- and downregulation hardly occur within the first three time points. Between hypg3 and 5 min, many downregulated genes become upregulated, and coherence is lower for upregulated genes and almost not present anymore for downregulated genes (i.e., initially downregulated genes do not stay downregulated). Between 5 min and hypg15, downregulated genes either remain downregulated or become unchanged, hardly any inversions are observed. For the upregulated genes, around ¼ remain upregulated, a minor fraction flips behavior. Additionally, previously not differentially expressed genes emerge as up-/downregulated. Therefore, coherence between 5 min and hypg15 is partly given. Highlighting only genes that were upregulated for hypg3 (Figure 9C) or hypg15 (Figure 9E) or downregulated for hypg3 (Figure 9D) or hypg15 (Figure 9F) allows in-depth characterization of groups of interest. Most of the upregulated genes at hypg3 (Figure 9C) are already upregulated at 75 s, while most of them are downregulated at hypg15. At 5 min, most of these genes do not appear differentially regulated. However, a fraction still remains upregulated, of which most genes no longer appear differentially regulated or are downregulated at 15 min. Therefore, overall coherence is given. On the other hand, for the genes that are downregulated at 3 min (Figure 9D), coherence is limited: A smaller fraction appears regulated in the same direction at 75 s, while at hypg15 most genes no longer appear differentially regulated. The opposite holds true for the hypg15 time point: upregulated genes (Figure 9E) are mostly not regulated for the early time points, only for 5 min, many genes already appear upregulated. For downregulated genes, however (Figure 9F), almost all initially upregulated genes at 20 s are covered, which holds also at 75 s. Only at 5 min, neutral and downregulated genes dominate fraction-wise. In general, two groups emerge: First, genes which are upregulated early on and which had a high coherence between 20 s, 75 s, and hypg3 timepoints, which then mostly become not differentially expressed at 5 min and downregulated at hypg15. Second, genes which are downregulated early on, some of which later become upregulated, but in general show significantly less coherence between the timepoints. These findings of coherence between different experimental platforms, particularly in real flight studies outside the lab environment, and between different analysis methods, suggest that the observed effects are a robust and biologically valid effect of hypergravity, despite the highly standardized and thus highly artificial biological system used in this study.

**FIGURE 10:**
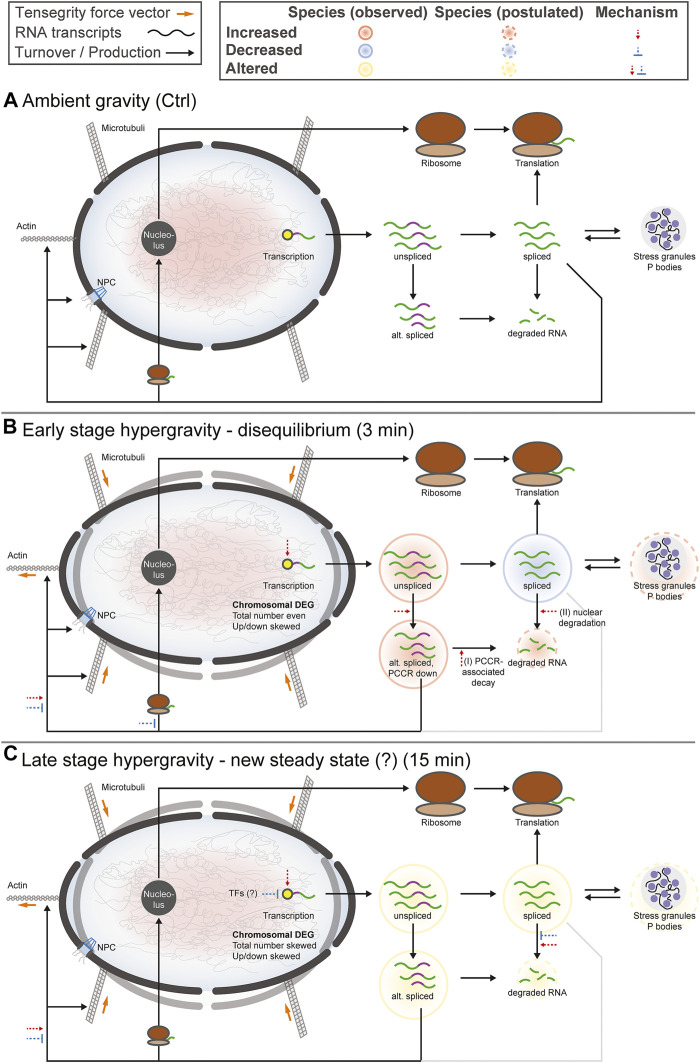
Summary of the transcriptional effects of hypergravity discovered in this study. Turnovers that are increased (red) and decreased (blue) by altered gravity are indicated by arrows. Effects that could be fully demonstrated are displayed as solid lines; effects that could be postulated are indicated by dashed lines. The placement of items does not necessarily reflect the location in the cell. For reasons of simplicity, not all connectors are shown, e.g., translation of alternatively spliced RNA transcripts. Alternatively spliced transcripts are composed of transcripts with and without altered PCCR, and include, but are not limited to, nucleolar-associated transcripts and transcripts from the cytoskeletal nuclear mechanical axis. **(A)** Steady state. **(B)** Early effects that could be detected at 3 min of hypergravity. **(C)** Late effects/long-lasting effects that could be detected after 15 min of hypergravity.

Taking the findings from this study together, and considering the coherence between similar hypergravity studies, this allows us to refine our model of reactions towards hypergravity (Figure 10). In the figure, details that could be proven and those that we could postulate are differentiated. In Figure 10A, the cell in its normal state in 1xg gravity is shown. From previous work, we hypothesized gravitational force-induced 3D conformational changes of the chromatin as primary driver behind transcriptional effects (Vahlensieck et al., 2021b), as indicated by the slightly squeezed nucleus, promoted by tensegrity-propagated force on the nuclear membrane. In the early phase (Figure 10B), transcription is increased by hypergravity-mediated conformation changes at different loci, distributed along all chromosomes. Only few genes are transcriptionally downregulated in this phase. In this point we can refine our initial model: Previously, we could not differentiate upregulation and downregulation and allocated both to conformational changes (Vahlensieck et al., 2021b). Based on this study, we can propose that initial upregulatory effects result from conformational changes of the chromatin as described, whereas initial downregulatory effects mostly result from active degradation. The newly emerging pre-mRNA is consecutively spliced. Depending on the model for PCCR effects, either 1) alternative splicing with retention of fragments that would be spliced out under normal gravity conditions is significantly elevated, which might be the case because complete splicing is impaired or 2) protein-coding transcripts are degraded in the nucleus since their export is limited by gravitational effects. In particular, an increased amount of noncoding mRNA appears (genes with PCCR down), which is preferably degraded. Since a fraction of PCCR down genes codes for nucleolar proteins, the formation of ribosomes is impeded, limiting protein translation overshoot. Furthermore, alternative splicing effects target the nuclear pore complex (NPC), actin and microtubule fibers. Generally, active RNA degradation is increased by hypergravity for many transcripts, acting on the spliced transcript pool only. Additionally, newly generated transcripts are likely scavenged by stress granules and P bodies. In total, these different regulatory levels likely limit the generation of proteins by mRNA translation, despite the elevated transcript levels for several genes.

In the late stage (Figure 10C), the system approaches a new steady state. Transcription is not only upregulated for some genes, but now also downregulated for other genes, likely by feedback loops that need some time to react, potentially including the transcription factors (TFs) DLX2, EMX1, and MSX1. The influence of alternative splicing is diminished, therefore also PCCR-associated degradation does not play a major role anymore, and nucleolar protein synthesis is no longer hindered on the level of transcripts. There are still transcripts with an increased rate of degradation, but for other genes the degradation of spliced mRNA is limited compared to normal gravity conditions. Stress granules and P bodies are likely also more balanced in term of scavenging and releasing transcripts. In total, this leads to more balanced translation, which is still different from normal gravity but no longer as skewed as in the early stage. This state either represents the new steady state or is approaching it.

To conclude, our refined model brings together the findings on the conformational origin of the transcriptional response to altered gravity and the composition and dynamics of the transcriptional response. By using mechanisms acting on different levels of the transcriptional and post-transcriptional machinery, the cell limits the broad, initial influence of altered gravity and brings the cell on track towards a new steady state.

For the future, to gain deeper insights into the characteristics of altered gravity-dependent differential transcription, an extended study with more time points could be beneficial, to better discriminate continuous differential expressing genes from very early or very late rebound behavior. Utilizing rRNA depletion libraries instead of polyA libraries for RNA-Seq would allow the analysis of partly transcribed/unfinished transcripts, thereby allowing a fine temporal resolution of early effects (Yang et al., 2011). A specific analysis of potential mediators of the rebound effect could generate explanations for the rapid adaption. In particular, miRNA is one regulatory factor to the transcript pool, facilitating mRNA degradation and mostly hindering translation, except for some cases where it activates translation (reviewed in (O'Brien et al., 2018)). Further, it is also considered an important player in buffering transcriptional noise (Siciliano et al., 2013; Schmiedel et al., 2015). miRNA-associated active degradation could be one driver of initial regulation, acting preferentially on the spliced pool, but not so much on the unspliced pool (O'Brien et al., 2018), which is in line with the observed transcript pool changes. Therefore, miRNA is a potential target for such studies. Additionally, to pinpoint the mechanism of mRNA degradation, a RNA decay analysis could be exploited (Chan et al., 2018; Ashworth et al., 2019). Further, the functional state of a cell is mostly determined by its protein content, for which mRNA content is not always a good predictor (Buccitelli and Selbach, 2020). Therefore, analyzing polysome (separating transcribing mRNA from non-transcribing) (Chassé et al., 2017; Poria and Ray, 2017) as a predictor of translation and using proteomics parallel to transcriptomics (Jiménez et al., 2022) of cells exposed to altered gravity have the potential to give a better understanding of downhill effects of differential gene expression.

## Data Availability

The datasets presented in this study can be found in online repositories. The names of the repository/repositories and accession number(s) can be found below: Gene Expression Omnibus accession number: GSE190484.
