# Supplementary material for: Post-Transcriptional Dynamics is Involved in Rapid Adaptation to Hypergravity in Jurkat T Cells
Source: Front Cell Dev Biol. 2022 Jul 4;10:933984. doi: 10.3389/fcell.2022.933984 (PMC9289288; doi:10.3389/fcell.2022.933984)
Supplement: Supplementary file 1 [file DataSheet1.pdf]

Supplementary Material

1 Supplementary Data

1.1 Supplementary Figures

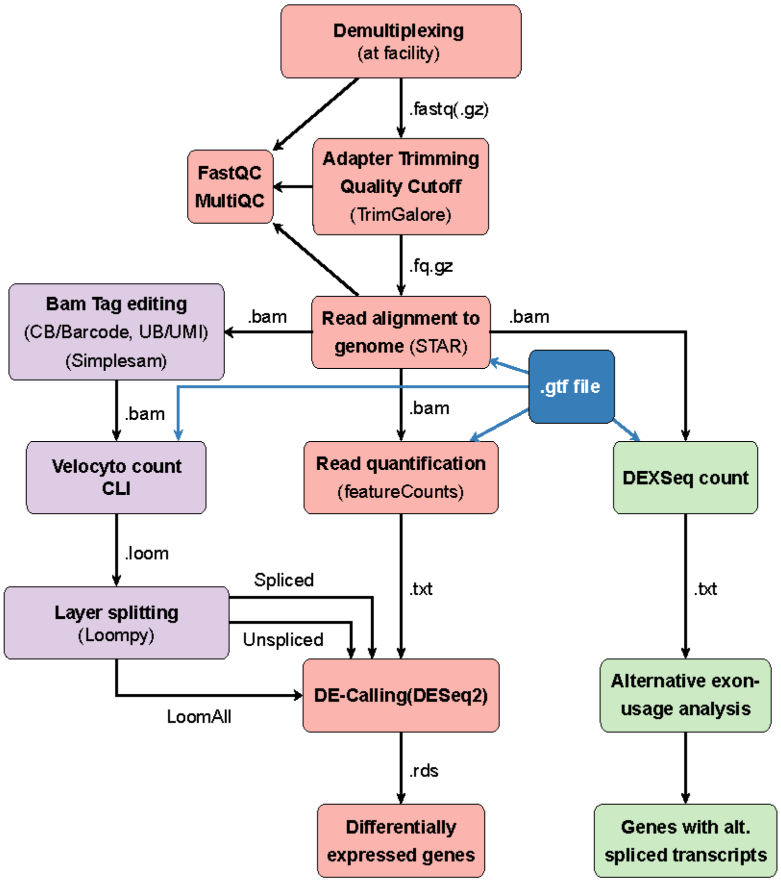

Supplementary Figure S1 – Analysis pipeline. The analysis pipeline consisted of a standard procedure (middle, red) with adapter & quality trimming of demultiplexed reads with TrimGalore/Cutadapt, read alignment with STAR mapper, counting of genes with featureCounts and differential expression analysis with DESeq2. Another analysis layer was added by splitting reads into spliced and unspliced fractions to gain a further information/temporal layer by distinguishing recently transcribed transcripts that did not have enough time to be spliced yet from already processed transcripts that had enough time for being processed. This was achieved by modifying aligned reads by adding one single cell UMI barcode to every read to prepare them for (single cell) RNA velocity analysis in Velocyto count. Afterwards, loom matrices were split into splicing layers (spliced, unspliced, LoomAll (all counts, no split between spliced and unspliced)) and the resulting count matrices fed back into the standard pipeline by evaluating them in DESeq2, layer by layer. Additionally, an alternative exon usage analysis was conducted on the aligned reads with the help of DEXSeq, a package that detects significant differences in exon usage between sequencing samples.

From a list of exons with significant differential usage, a list of genes with alternatively spliced transcripts was generated.

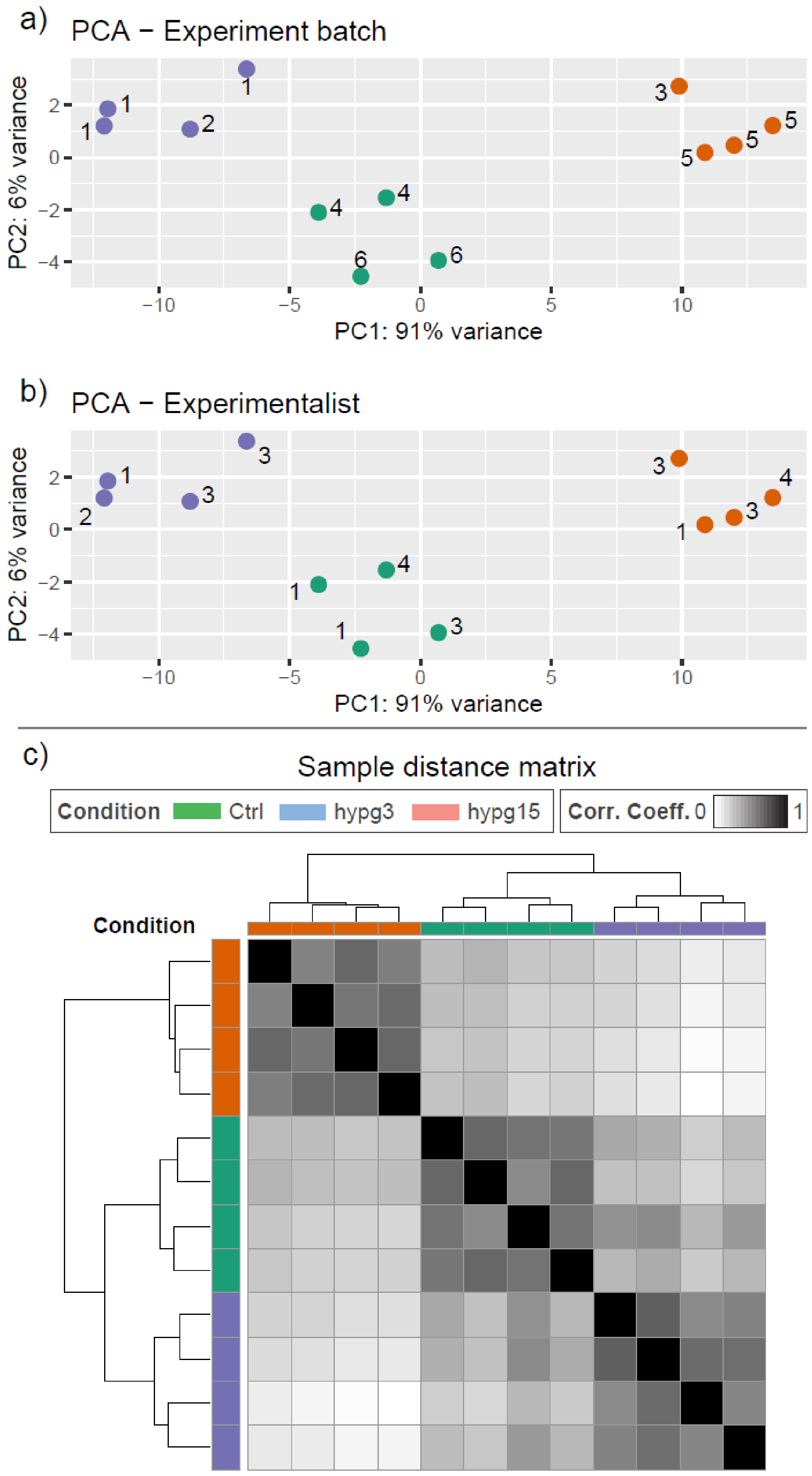
a)
PCA − Experiment batch
PC2: 6% variance
PC1: 91% variance
b)
PCA − Experimentalist
PC2: 6% variance
PC1: 91% variance
c)
Sample distance matrix
Condition
Ctrl
hypg3
hypg15
Corr. Coeff. 0
1
Condition

Supplementary Figure S2 – Grouping analysis of sequencing data. **(a)** PCA plot of normalized count data for all twelve samples in the dataset. Samples were taken in batches of four due to experimental restrictions. The experiment batch is indicated for each sample point. No fundamental batch effect could be detected for the experiment batch. **(b)** Same PCA plot as in (a). It was tested if the experimentator who took care of a certain sample could be a confounder of sample clustering. The (anonymized) experimentator is annotated for all samples. No experimentator-related batch effect could be detected. **(c)** Sample distance matrix for normalized count data. Correlation coefficient between samples is given between 0 (no correlation) and 1 (perfect correlation), the identity (sample vs itself, correlation coefficient of 1) is in the diagonal. All three experimental conditions sharply cluster. The samples from the group Ctrl and hypg3 show some correlation with each other.

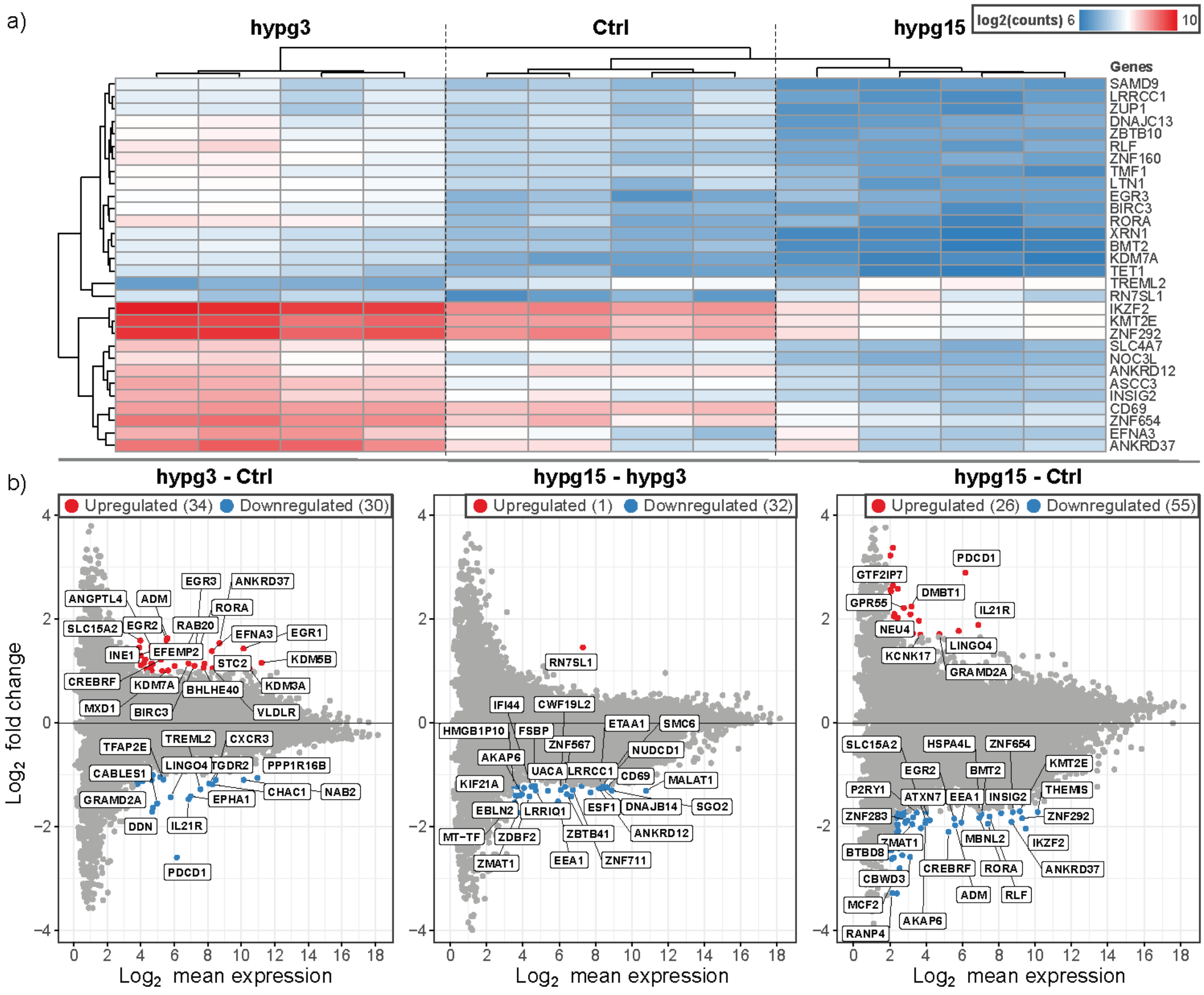

Supplementary Figure S3 – Extended characterization of effects dynamics. **(a)** Clustered heatmap of counts of the top 30 genes with maximum variation in counts. Genes with counts under 50 have been excluded to avoid shot noise. **(b)** MA plots of all three temporal comparisons show the overall distribution. Genes with highest fold change that exceed a certain mean expression threshold are annotated. Hypg15-hypg3 and hypg15-Ctrl show a pronounced skew towards downregulation, for hypg3 a slight tendency towards upregulation can be observed for the fold changes.

	GO biological process complete	Total	In set	expected	enriched	+/-	P value
Upregulated	dendrite development	111	10	1.23	8.1	+	0.00855
	↳ neuron projection development	656	24	7.3	3.29	+	0.00496
	↳ neuron development	819	29	9.11	3.18	+	0.00058
	↳ cell development	1668	40	18.55	2.16	+	0.0432
	↳ neuron differentiation	1018	31	11.32	2.74	+	0.00494
	↳ generation of neurons	1081	32	12.02	2.66	+	0.0058
	↳ neurogenesis	1223	33	13.6	2.43	+	0.0329
	↳ nervous system development	2081	49	23.15	2.12	+	0.00452
	cell morphogenesis involved in neuron differentiation	422	19	4.69	4.05	+	0.00432
	↳ cell morphogenesis involved in differentiation	535	22	5.95	3.7	+	0.00232
	↳ cell morphogenesis	686	26	7.63	3.41	+	0.00083
	↳ anatomical structure morphogenesis	2185	48	24.3	1.98	+	0.0436
	neuron projection morphogenesis	462	20	5.14	3.89	+	0.00387
	↳ plasma membrane bounded cell projection morphogenesis	466	20	5.18	3.86	+	0.0044
	↳ cell projection morphogenesis	470	20	5.23	3.83	+	0.00501
	↳ cell part morphogenesis	489	20	5.44	3.68	+	0.00904
	chromatin organization	590	22	6.56	3.35	+	0.0112
	positive regulation of nitrogen compound metabolic process	3142	63	34.95	1.8	+	0.0277
Downregulated	proton motive force-driven mitochondrial ATP synthesis	64	6	0.23	25.74	+	0.00187
	↳ proton motive force-driven ATP synthesis	73	6	0.27	22.56	+	0.00387
	↳ ATP biosynthetic process	85	6	0.31	19.38	+	0.00896
	↳ cellular nitrogen compound metabolic process	3354	30	12.22	2.46	+	0.00807
	↳ organonitrogen compound biosynthetic process	1331	18	4.85	3.71	+	0.0103
	↳ purine ribonucleoside triphosphate biosynthetic process	96	6	0.35	17.16	+	0.0175
	↳ purine nucleoside triphosphate biosynthetic process	97	6	0.35	16.98	+	0.0186
	↳ nucleoside triphosphate biosynthetic process	114	6	0.42	14.45	+	0.0452
	↳ purine nucleoside triphosphate metabolic process	116	6	0.42	14.2	+	0.0497
	↳ ribonucleoside triphosphate biosynthetic process	102	6	0.37	16.15	+	0.0245
	↳ purine ribonucleoside triphosphate metabolic process	111	6	0.4	14.84	+	0.039
	↳ aerobic respiration	158	7	0.58	12.16	+	0.0211
	mitochondrial respiratory chain complex I assembly	60	5	0.22	22.88	+	0.0353
	↳ NADH dehydrogenase complex assembly	60	5	0.22	22.88	+	0.0353
	↳ mitochondrial respiratory chain complex assembly	95	6	0.35	17.34	+	0.0166

Supplementary Figure S4 – Gene Ontology analysis of continuously upregulated and downregulated genes from Figure 1. Target sets from GO Biological Process, analyzed by Gene Ontology PANTHER17.0. Larger sets that contain the previous set are indicated by an arrow. Table columns are: Set name, total number of contained genes in a set, number of genes from a set that are continuously regulated, expected number of continuously regulated genes to be included in a set by random drawing, enrichment coefficient (division of the previous two numbers), over- versus under-enrichment, Bonferroni-corrected P value.

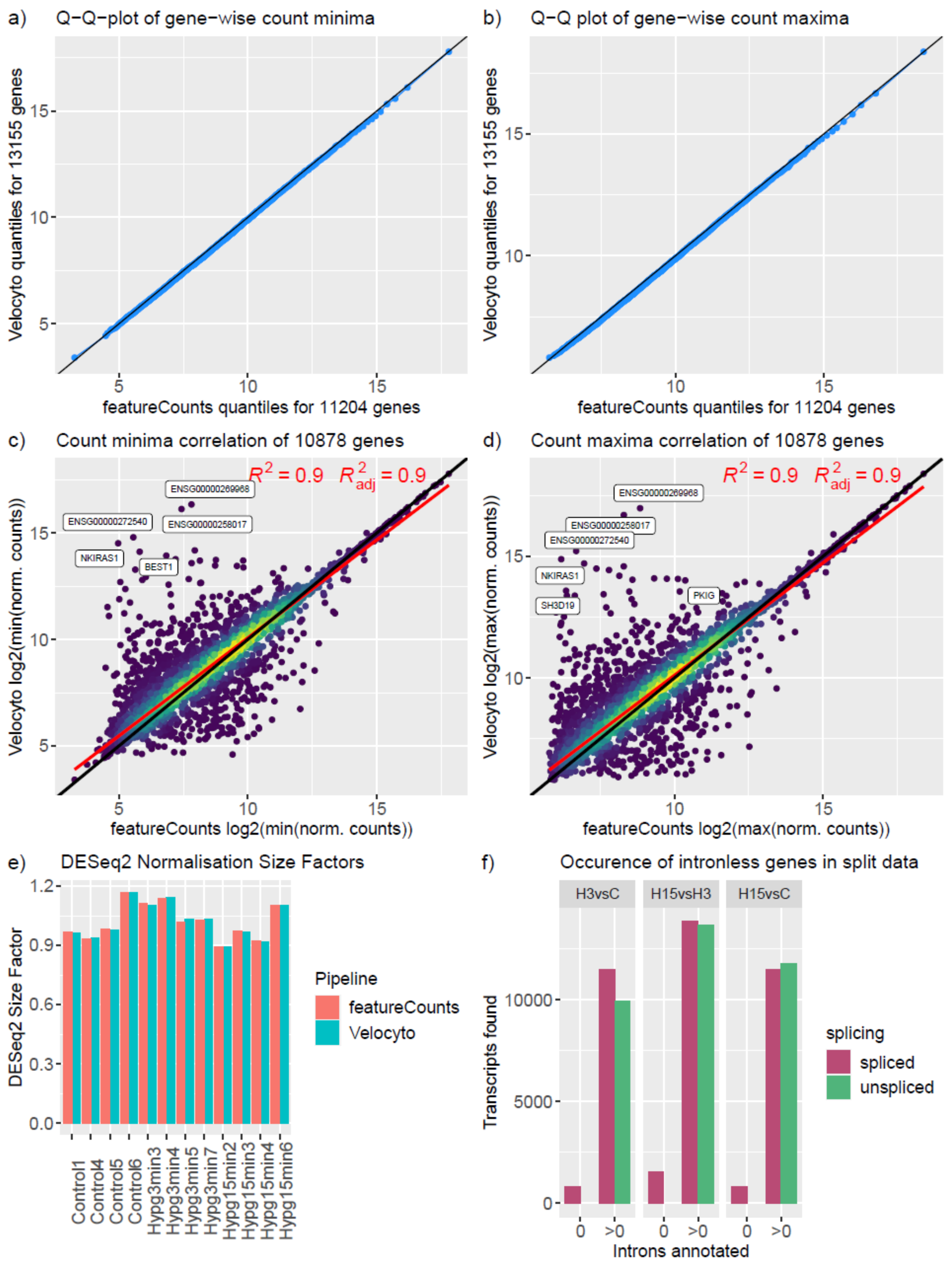

a) Q−Q−plot of gene−wise count minima
Velocyto quantiles for 13155 genes
featureCounts quantiles for 11204 genes
b) Q−Q plot of gene−wise count maxima
Velocyto quantiles for 13155 genes
featureCounts quantiles for 11204 genes
c) Count minima correlation of 10878 genes
$R^2 = 0.9$ $R^2_{adj} = 0.9$
ENSG00000269968
ENSG00000272540
ENSG00000258017
NKIRAS1
BEST1
Velocyto log2(min(norm. counts))
featureCounts log2(min(norm. counts))
d) Count maxima correlation of 10878 genes
$R^2 = 0.9$ $R^2_{adj} = 0.9$
ENSG00000269968
ENSG00000258017
ENSG00000272540
NKIRAS1
PKIG
SH3D19
Velocyto log2(max(norm. counts))
featureCounts log2(max(norm. counts))
e) DESeq2 Normalisation Size Factors
DESeq2 Size Factor
Pipeline
featureCounts
Velocyto
Control1
Control4
Control5
Control6
Hypg3min3
Hypg3min4
Hypg3min5
Hypg3min7
Hypg15min2
Hypg15min3
Hypg15min4
Hypg15min6
f) Occurence of intronless genes in split data
H3vsC
H15vsH3
H15vsC
Transcripts found
splicing
spliced
unspliced
Introns annotated

Supplementary Figure S5 – Comparative analysis and validation of modified Velocyto-on-bulk pipeline for count data. **(a,b)** Comparisons of quantiles for normalized log2-transformed gene count minima/maxima over all samples for featureCounts and Velocyto. A black line indicates the expected distribution if gene counts were in perfect match between the two pipelines. **(c,d)** log2-transformed normalized count minima/maxima over all samples for featureCounts and Velocyto. The color coding represents density of features, yellow areas contain large numbers of genes. The black line represents a perfect overlap between both methods, the red line the actual linear fit. The coefficient of determination (R^2) is given, indicating a strong agreement. Single genes that deviated strongly are annotated. Genes with mean counts below 50 have been excluded to remove shot noise. **(e)** Normalization factors from DESeq2 for all twelve samples for the featureCounts and Velocyto pipeline. A large deviation between the two values for one sample would indicate a structural difference between counting datasets for the classical featureCounts pipeline and the Velocyto pipeline. **(f)** Number of annotated introns for genes in different layers in the Velocyto dataset to test for correct annotation. Genes with one intron or more are part of both spliced and unspliced datasets. Genes without annotated introns only appear in the spliced dataset as it would be expected by definition, since unspliced genes should at least carry one intron.

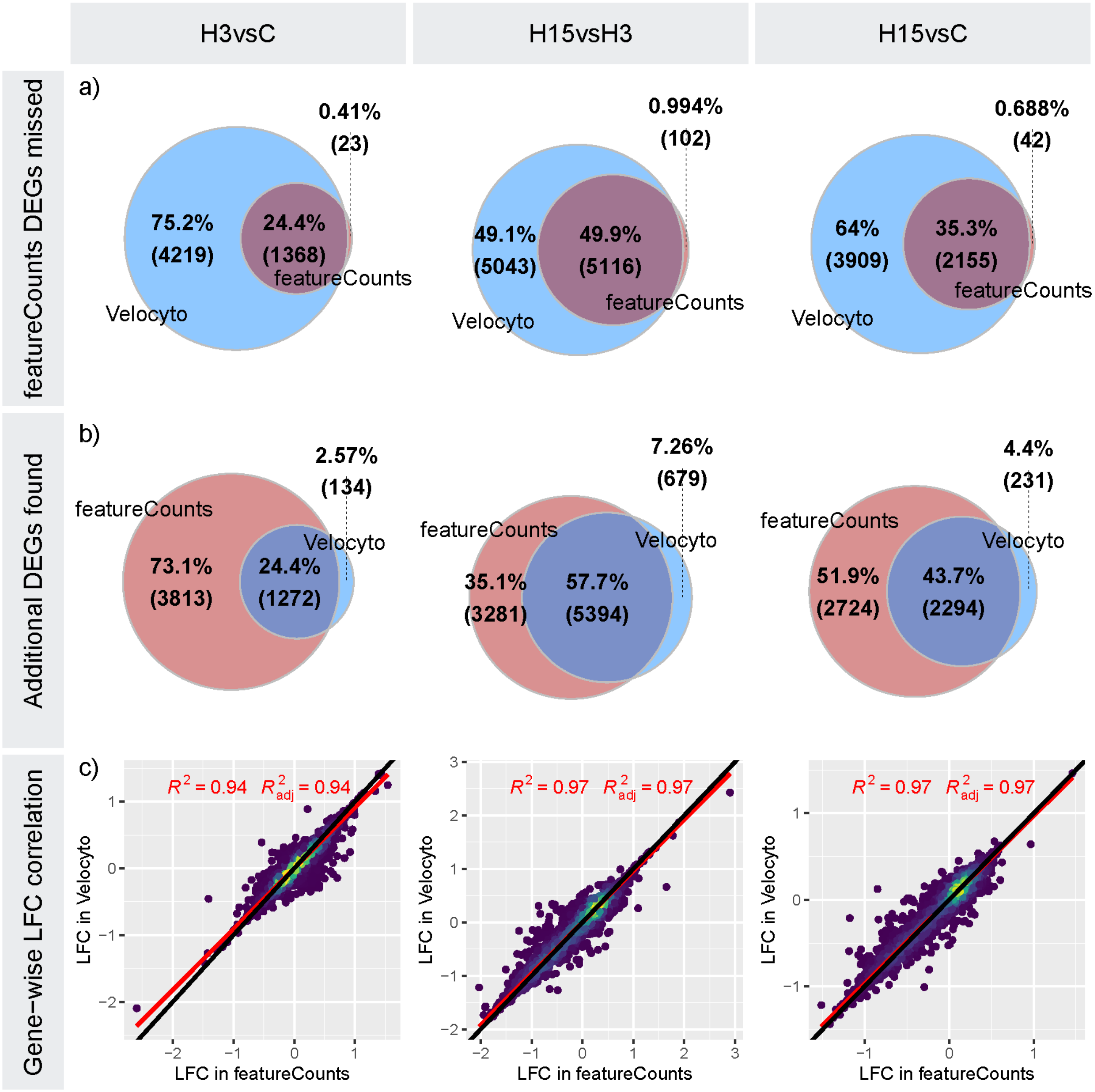

Supplementary Figure S6 – Velocyto-on-bulk pipeline validation for comparisons. Correlation between featureCounts pipeline and custom Velocyto pipeline for all three temporal comparisons. **(a)** Differentially expressed genes in the featureCounts sets that were not present in the Velocyto all sets ("DEG missed"). DEGs for featureCounts were selected by a strict cutoff (padj < 10^{-4}) and DEGs for Velocyto by a more lose cutoff (padj < 0.05) to be able to identify highly significant DEGs in the featureCounts set that were not present in the Velocyto even at lower significance levels. The percentage of such is very small compared to the overlap. **(b)** Additional highly significant DEGs found in the Velocyto set (padj < 10^{-4}) that were not present in the featureCount set even at less restrictive cutoff (padj < 0.05). Velocyto identified additional genes as differentially expressed compared to featureCounts. These have been identified to consist partly of antisense transcripts since

Velocyto did not differentiate between forward and antisense transcripts as well as featureCounts did, but the overall effect was minor compared to the high agreeability between the methods. **(c)** Comparison of log fold change (LFC) reported for Velocyto and for featureCounts. Genes with a mean count below 50 have been excluded to suppress shot noise. A black line represents perfect correlation without any deviation, the red line shows a linear fit for actual correlation. The coefficient of determination (R^2) is given that represents the goodness of fit.

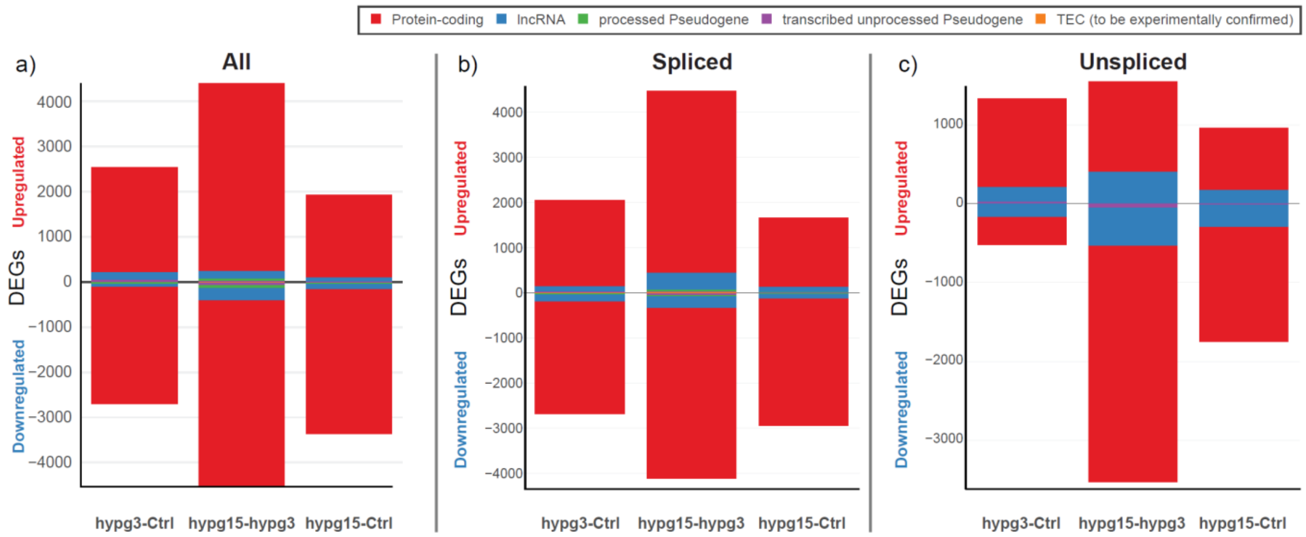

Supplementary Figure S7 – **(a)** Ensembl biotype of differentially expressed genes for all three comparisons. Protein-coding genes were the dominant type, lncRNA shows a relatively increased fraction for downregulation in the hypg15-hypg3 fraction. A limit of a minimum of 20 differentially expressed genes in total for a given gene biotype was added to exclude groups of transcripts with very low abundance. **(b/c)** Ensembl biotype of differentially expressed genes, split by spliced and unspliced layer. Absolute numbers could not be compared between unspliced and spliced pools due to the different pools of genes that can be detected in the spliced and unspliced fraction.

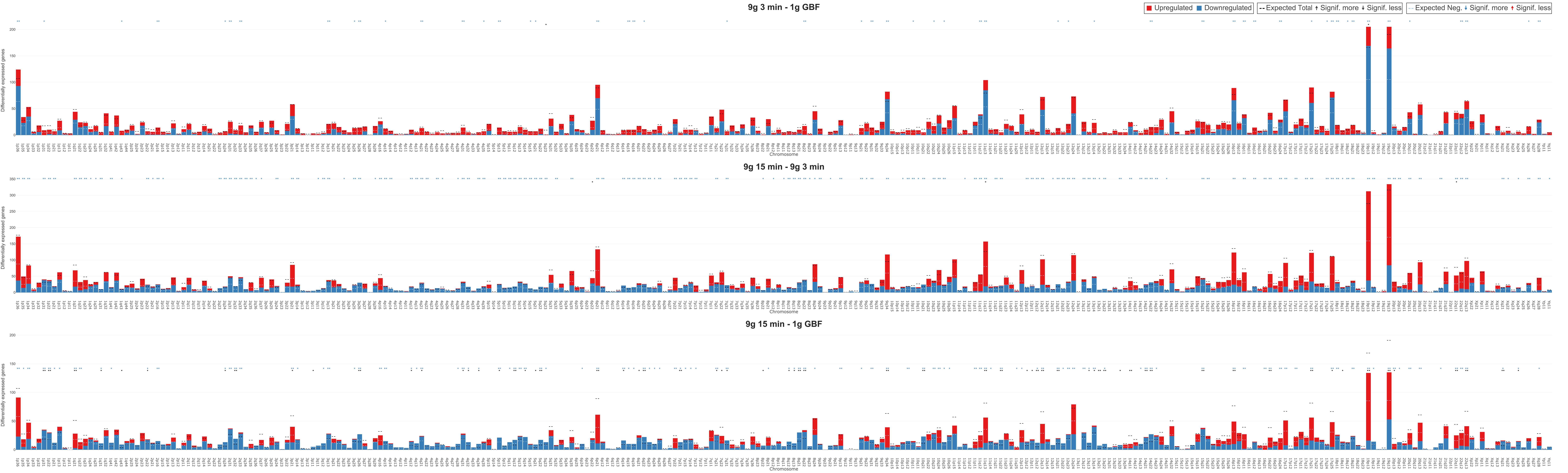

9g 3 min - 1g GBF
Upregulated
Downregulated
Expected Total
Signif. more
Signif. less
Expected Neg.
Signif. more
Signif. less
Differentially expressed genes
Chromosome
9g 15 min - 9g 3 min
Differentially expressed genes
Chromosome
9g 15 min - 1g GBF
Differentially expressed genes
Chromosome

Supplementary Figure S8 – Chromosomal distribution of differential gene expression – cytoband level. Number of differentially upregulated (red) and downregulated (blue) genes per chromosome cytoband (X axis) for all three comparisons. Bands are ordered by their appearance in karyograms. The expected number (if there was no selection towards certain bands) of total DEGs per band (based on the fraction of differentially expressed genes for all genes and the number of detected genes per band) is shown as black dashed line, the expected number of upregulated genes out of up- and downregulated genes is shown as dashed light red line. If the actual number significantly (tested by an FDR-corrected Fisher test) differs from the expected value, this is indicated by a (*) for P<.05 and (**) for P<.01.

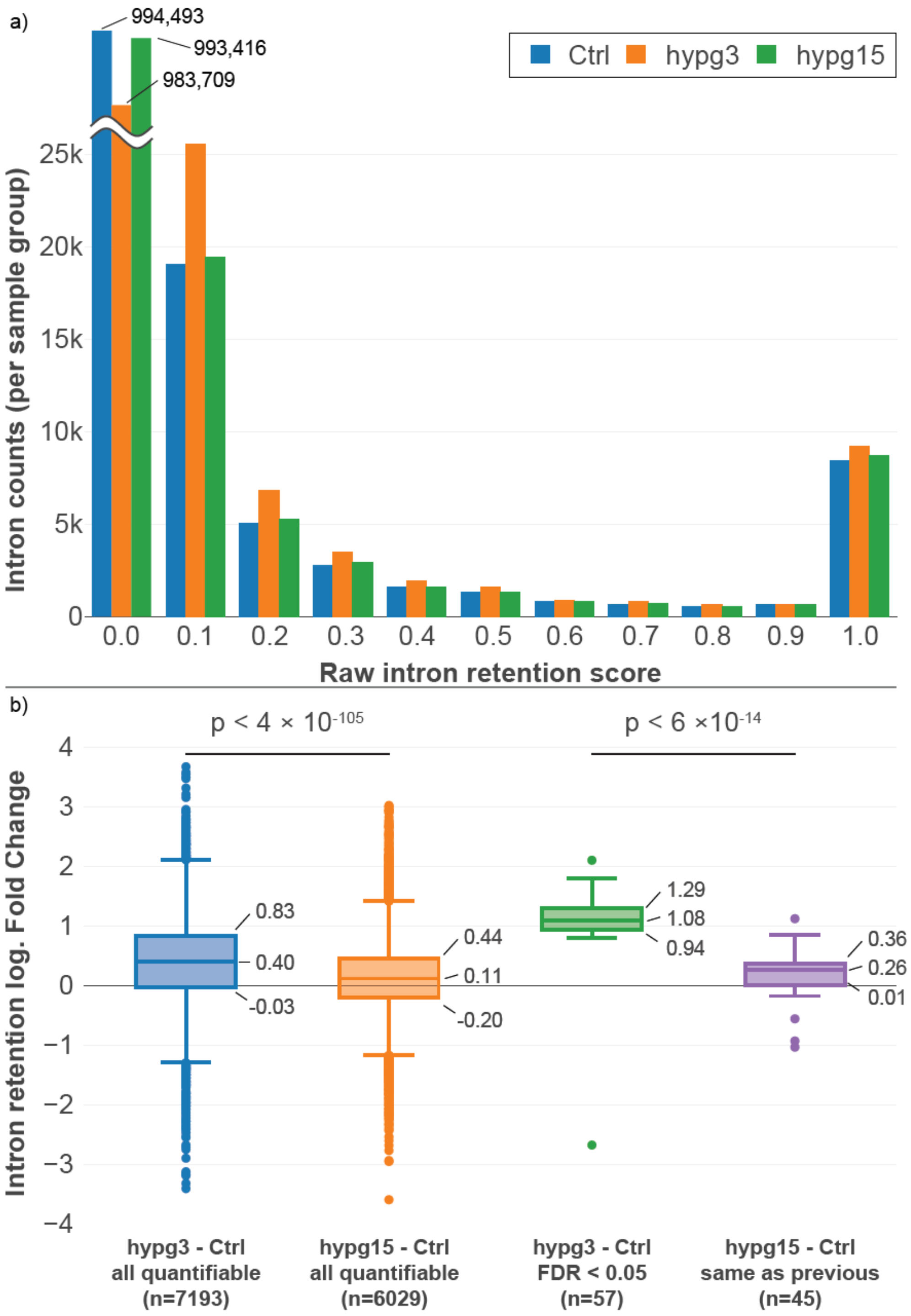

Supplementary Figure S9 – Quantitative intron retention rate analysis. **(a)** Histogram of all identified introns that could be detected per sample group, stratified by its raw intron retention score, binned in steps of 0.1. No averaging between the four replicates in a sample group has been performed, therefore one intron can appear up to four times per sample group. **(b)** Changes in intron retention for two comparisons as box plots for all quantifiable exons (first two boxes), only for those that are significantly changed for hypg3-Ctrl (third box), and the fold changes for the comparison

hypg15-Ctrl for the introns that showed significantly altered intron retention for hypg3-Ctrl (fourth box). A positive log. fold change (vertical axis) corresponds to an increase in intron retention. To test if distributions were significantly different for different comparisons, a t test was applied between the two overall distributions for different comparisons, and for the two distributions of introns that are significantly differentially retained after three minutes. p values are listed above.

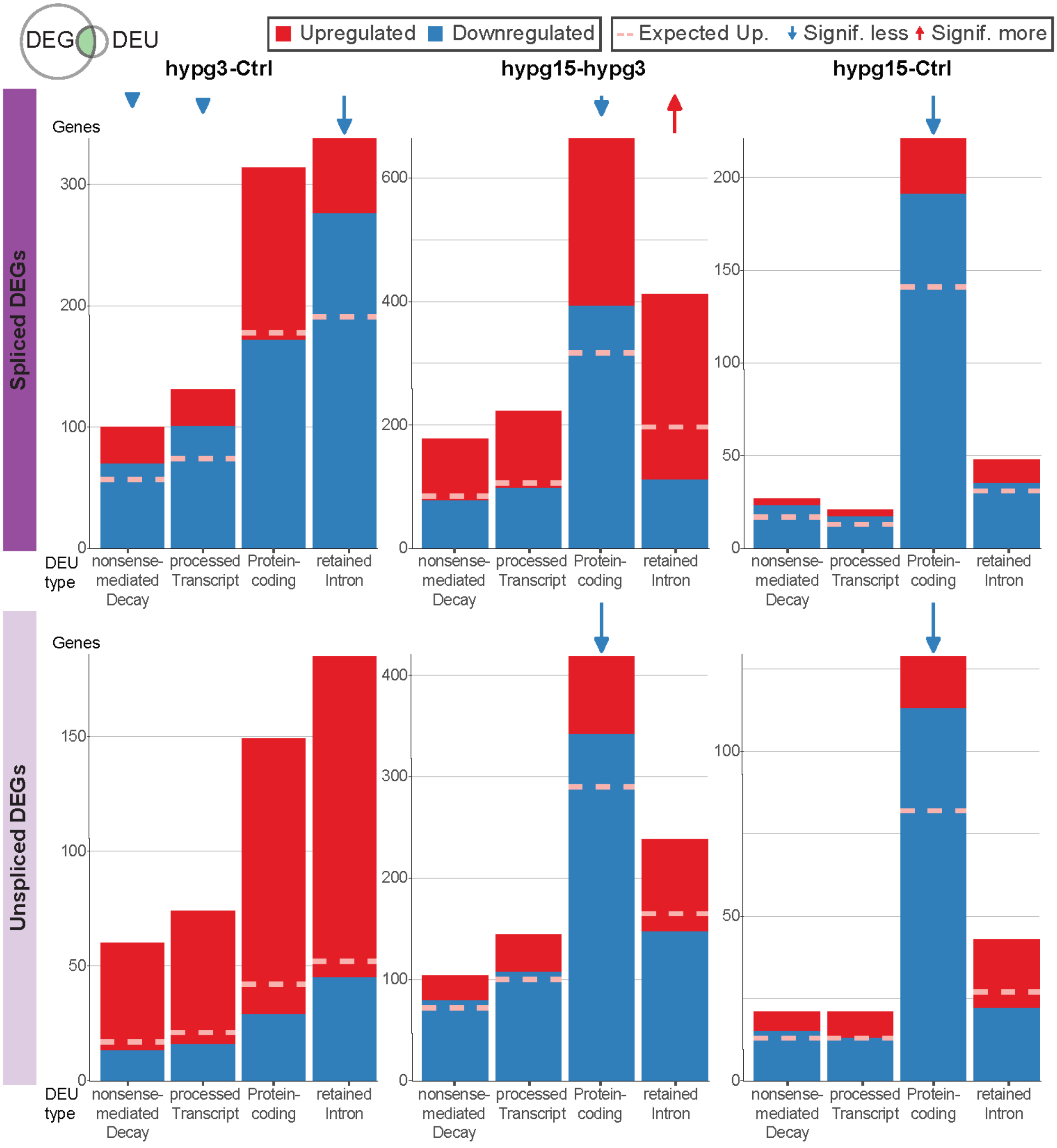

Supplementary Figure S10 – Characterization of genes that showed differential expression and differential exon usage – unspliced data, addition to figure 5c. Plotting of upregulated (red) & downregulated (blue) differentially expressed genes that also underwent differential exon usage for all three temporal contrasts. For the DEGs, the unspliced and the spliced pool are shown separately. Per definition the unspliced pool should not be informative about alternative splicing. Therefore, effects that parallelly appear in both unspliced and spliced data should not be an effect of alternative splicing but appear in advance. Data is split by DEU exon biotype on the x axis. The expected fraction of upregulated DEGs (based on fraction of upregulated vs downregulated genes for all genes multiplied with number of DEG DEU overlaps with the exon biotype specified) is indicated by a dashed line. If the actual value of upregulated genes in comparison to downregulated genes

significantly differs from expectation, this is indicated by an arrow on top (more than expected: arrow pointing upwards).

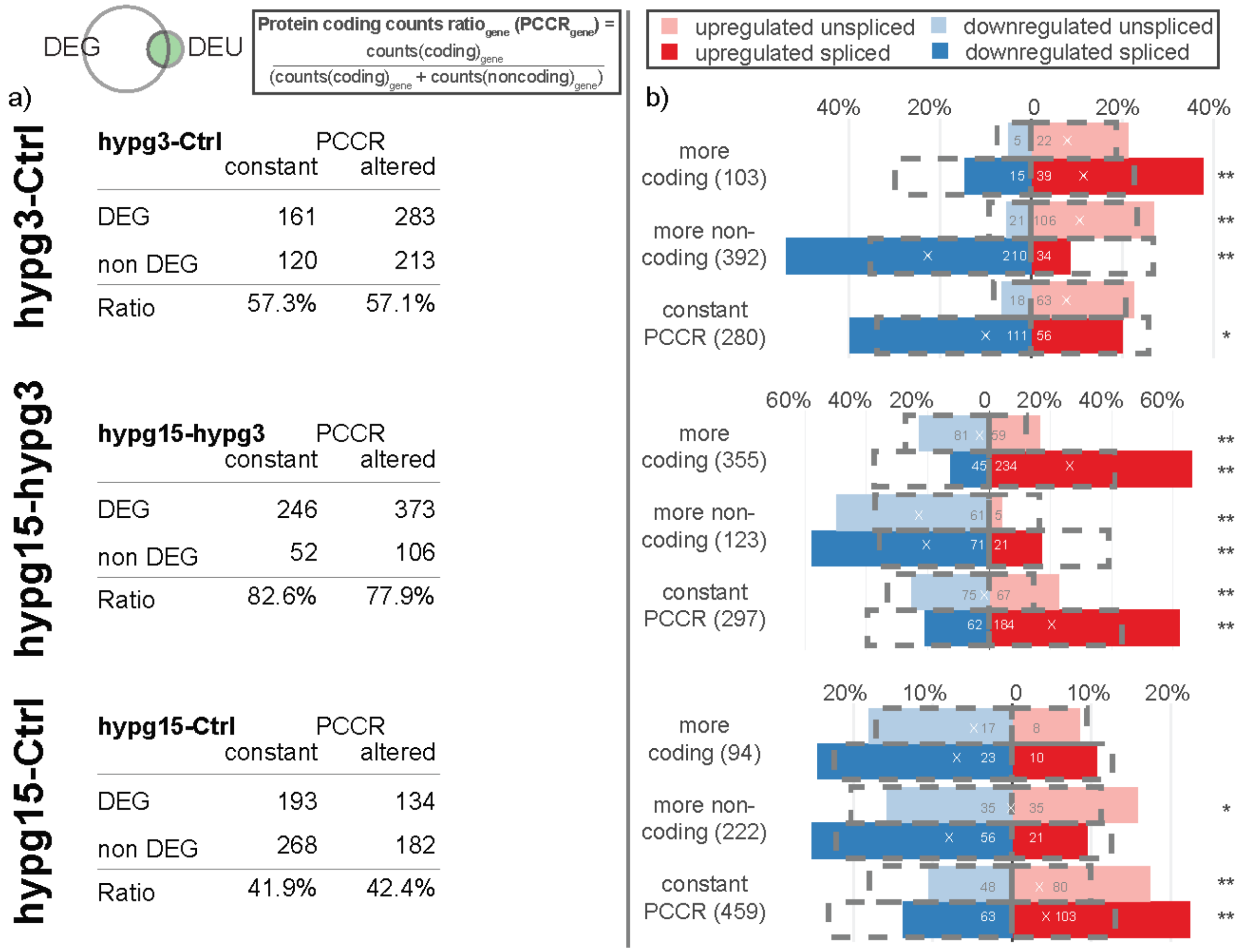

Supplementary Figure S11 – Protein Coding Counts Ratio for all comparisons. Extension of Figure 6. **(a)** The fraction of differentially expressed genes was analyzed for genes where the PCCR was altered (up and down) and for those not altered. **(b)** Split between genes with constant PCCR, increased PCCR, and decreased PCCR. For each subset of genes, the number of genes that is significantly upregulated or downregulated in the spliced and unspliced fraction was indicated. The grey boxes indicate the expected distribution from the overall DEG datasets. Significant deviations from expectation based on a Fisher exact test are indicated (** for $p < 0.01$, * for $p < 0.05$). An “X” indicates the mean direction of differential gene expression.

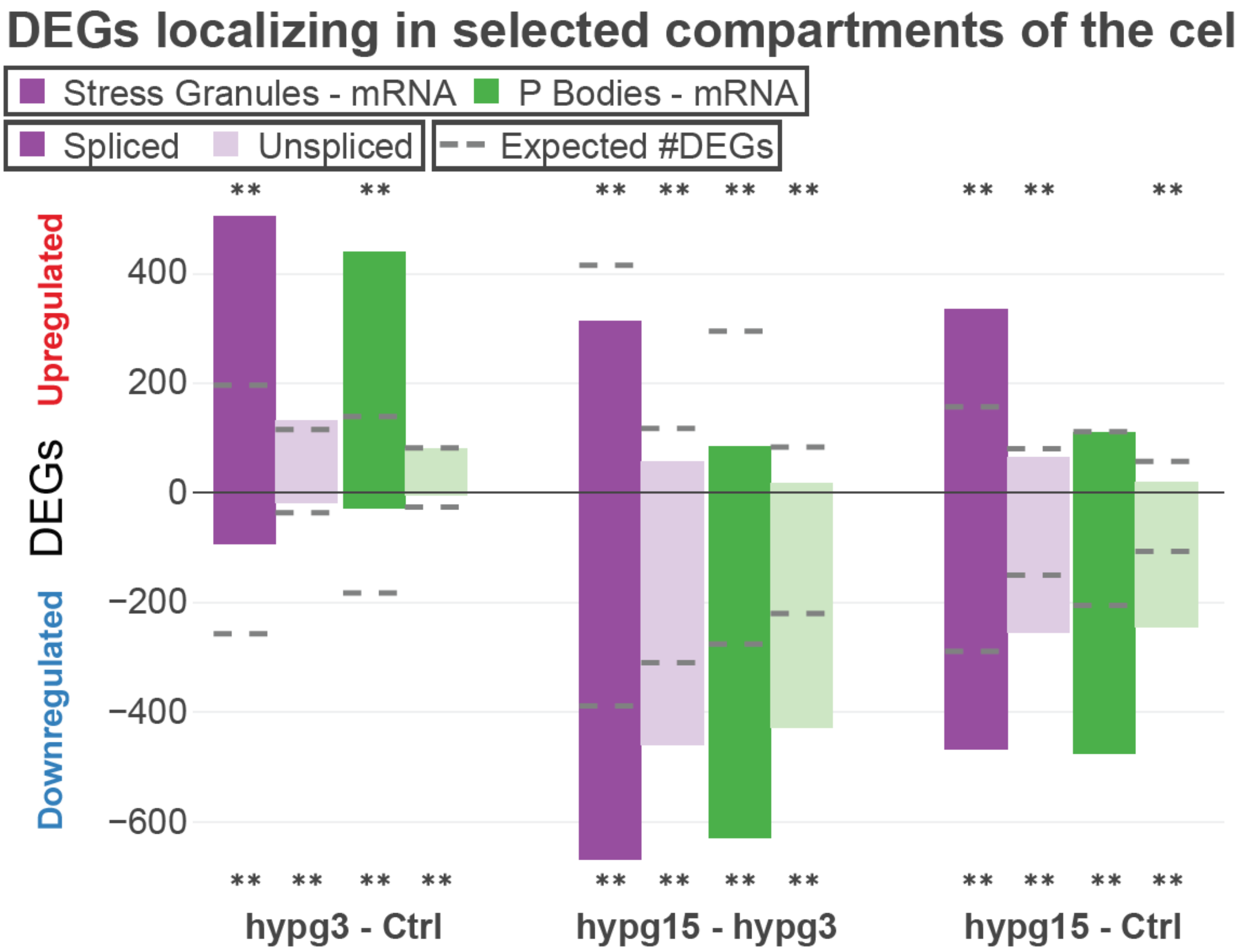

Supplementary Figure S12 - Cellular compartments that are involved in transcript pool regulation following stress responses were assessed for their association with the differential expression patterns for all three temporal comparisons. For stress granules and P bodies, RNA-Seq datasets of their mRNA content were intersected with differential gene expression due to altered gravity. The actual and the expected number of upregulated and downregulated genes in the hypergravity dataset for transcripts that show affinity for stress granules / P bodies is shown. After 3 minutes, the number of upregulated genes in the spliced pool that show affinity towards the tested compartments significantly exceeds the expectation, while the number of downregulated genes is far less than expected. This does not hold true for unspliced genes.
